# Structural basis for genome wide recognition of 5-bp GC motifs by SMAD transcription factors

**DOI:** 10.1038/s41467-017-02054-6

**Published:** 2017-12-12

**Authors:** Pau Martin-Malpartida, Marta Batet, Zuzanna Kaczmarska, Regina Freier, Tiago Gomes, Eric Aragón, Yilong Zou, Qiong Wang, Qiaoran Xi, Lidia Ruiz, Angela Vea, José A. Márquez, Joan Massagué, Maria J. Macias

**Affiliations:** 1 Institute for Research in Biomedicine (IRB Barcelona), The Barcelona Institute of Science and Technology, Baldiri Reixac, 10, 08028 Barcelona, Spain; 2 EMBL Grenoble, 71 Avenue des Martyrs, CS 90181, 38042 Grenoble, Cedex 9 France; 3 Cancer Biology and Genetics Program, Memorial Sloan Kettering Cancer Center, New York, NY 10065 USA; 4 Gerstner Sloan Kettering Graduate School of Biomedical Sciences, Memorial Sloan Kettering Cancer Center, New York, NY 10065 USA; 5 ICREA, Passeig Lluís Companys 23, 08010 Barcelona, Spain; 6 Present Address: Center for the Science of Therapeutics, Broad Institute of MIT and Harvard , 415 Main St, Cambridge, MA 02142 USA; 7 Present Address: MOE Key Laboratory of Protein Sciences, School of Life Sciences, Tsinghua University, Beijing, 100084 China

## Abstract

Smad transcription factors activated by TGF-β or by BMP receptors form trimeric complexes with Smad4 to target specific genes for cell fate regulation. The CAGAC motif has been considered as the main binding element for Smad2/3/4, whereas Smad1/5/8 have been thought to preferentially bind GC-rich elements. However, chromatin immunoprecipitation analysis in embryonic stem cells showed extensive binding of Smad2/3/4 to GC-rich *cis*-regulatory elements. Here, we present the structural basis for specific binding of Smad3 and Smad4 to GC-rich motifs in the goosecoid promoter, a nodal-regulated differentiation gene. The structures revealed a 5-bp consensus sequence GGC(GC)|(CG) as the binding site for both TGF-β and BMP-activated Smads and for Smad4. These 5GC motifs are highly represented as clusters in Smad-bound regions genome-wide. Our results provide a basis for understanding the functional adaptability of Smads in different cellular contexts, and their dependence on lineage-determining transcription factors to target specific genes in TGF-β and BMP pathways.

## Abstract (web summary)

Smad transcription factors are part of the TGF-β signal transduction pathways and are recruited to the genome by cell lineage-defining factors. Here, the authors identify specific Smad binding GC-rich motifs and provide structural information showing Smad3 and Smad4 bound to these motifs.

## Introduction

The transforming growth factor β (TGF-β) family of cytokines regulates critical processes during the lifecycle of metazoans, with important roles during embryo development, tissue homeostasis, regeneration, and immune regulation^1^. The main TGF-β signal transduction mechanism involves Smad transcription factors, and mutations in the components of this pathway are responsible for various inherited and somatic diseases^2–4^. The receptors for TGF-β, nodal, activin, myostatin, and other family members are membrane serine/threonine kinases that phosphorylate and activate Smad2 and Smad3, whereas analogous receptors for the bone morphogenetic proteins (BMPs) preferentially phosphorylate and activate Smads 1, 5, and 8. These receptor-activated Smads (R-Smads) form heterotrimeric complexes with Smad4, which is required for the transcriptional regulation of most target genes^5^. The Smad complexes are recruited to sites throughout the genome by cell lineage-defining transcription factors (LDTFs) that determine the context-dependent nature of TGF-β action. The first identified member of this class of Smad partners is FoxH1 (previously known as FAST1), which binds to nodal-activated Smad2/3–Smad4 complexes to regulate the expression of goosecoid (*Gsc*) and other mesendoderm differentiation genes in early-stage embryos^6–8^. The interaction of Smad2/3–Smad4 with FoxH1 in mesendoderm progenitors, and with the LDTFs MyoD in myoblasts, PU.1 in B-cell progenitors, GATA1 in erythroid progenitors, and C/EBPα in myeloid progenitors^9, 10^, represent a paradigm for the versatile, context-dependent regulation of cell stage transitions by TGF-β family factors.

Smad proteins consist of an N-terminal MH1 domain, which binds to DNA, a linker region with phosphorylation sites for network regulatory inputs, and a C-terminal MH2 domain that is phosphorylated by the receptors and binds other Smad proteins, LDTFs, chromatin readers, and transcriptional co-activators and co-repressors^3, 4^. Early insights into the DNA-binding specificity of Smad proteins came from oligonucleotide binding screens, which identified the palindromic duplex 5′-GTCTAGAC-3′ as a high-affinity binding sequence for Smad3 and Smad4^11^. The X-ray crystal structure of this sequence bound to MH1 domains of Smads 1, 3, 4, and 5 showed that the MH1 domain recognizes the GTCT motif. GTCT or its complementary extended sequence are referred to as the CAGAC Smad binding element (SBE)^12–15^. Subsequently Smad1 and Smad5 were shown to also recognize GC-rich motifs, termed BMP response element (BRE), in certain BMP-responsive genes^16–19^. This apparent dichotomy of SBEs in the TGF-β vs. BMP pathways was surprising, given the extensive sequence and structural similarity of the DNA-binding β-hairpin across Smad MH1 domains. Moreover, DNase I footprinting analysis of the *Gsc* promoter in vitro^19, 20^ and genome-wide ChIP-Seq experiments in live cells^9, 21–24^ showed that Smad3 and Smad4 can also bind to GC-rich regions lacking CAGAC sequences.

The mounting evidence that CAGAC may not be the only, or even the strongest Smad-binding element in TGF-β target genes led us to investigate the interaction of Smad3 and Smad4 with GC-rich DNA sequences. Focusing on the human and mouse *Gsc* promoter, here we identify specific Smad-binding GC-rich motifs and provide the X-ray crystal structures of Smad3 and Smad4 bound to several of these motifs. Smad1, Smad5, and Smad8 also bind to these motifs. A high degree of conformational flexibility of the β-hairpin allows recognition of different 5-bp GC-rich sequences. Functional assays confirmed that these promoter sites are required for the *Gsc* response to nodal in embryonic stem (ES) cells. The X-ray crystal structure of *Trichoplax adhaerens* Smad4 MH1 bound to one of these motifs indicates a high conservation of this interaction in metazoans. Based on these insights we delineated a consensus GGC(GC)|(CG) SBE for GC sites, which we refer to as the 5GC SBE. Clusters of 5GC SBEs are significantly enriched in Smad-binding *cis*-regulatory elements (CREs) of many TGF-β target genes, and are more prevalent in Smad target sites than is the CAGAC motif.

## Results

### Nodal-driven Smads bind to GC regions for *Gsc* activation

**Fig. 1:**
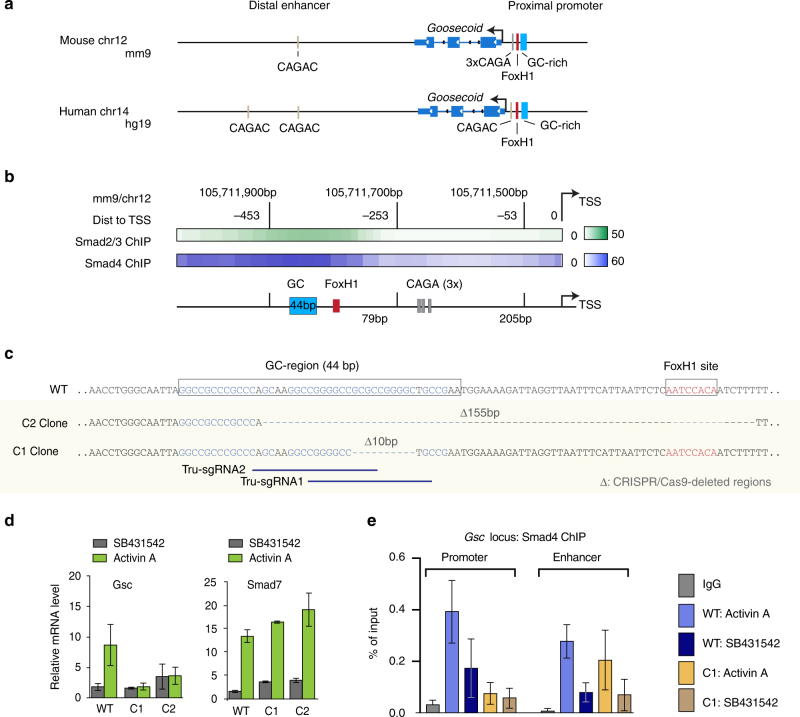
Motif identification in *Gsc* promoter using ChIP-Seq and CRISPR/Cas9. **a** Schematic representation of the proximal promoter and distal enhancer sites of mouse and human *Gsc* showing the GC-rich region, CAGAC, and the FoxH1 binding sites. **b** Heatmap of ChIP-Seq tag densities for Smad2/3 (GSM1782914^62^) and Smad4 (GSM2746361, this work) located at −600 bp from the TSS of Gsc, showing how the signal is centered at the GC-rich region. Coordinates referred to the mm9 genome assembly. Data are displayed for chromosome 12 between 105,711,900 and 105,711,700 bases. **c** Scheme of the CRISPR/Cas9-mediated mutagenesis of the *Gsc* proximal promoter region. CRISPR-mediated deletions of a 10 bp region of the *Gsc* GC1 site (Clone C1) and of the GC1-FoxH1 region (155 bp, Clone C2) are indicated with dashed lines. The DNA sequence of targeted regions is represented as blue horizontal bars. Deletions were confirmed by deep sequencing and TA cloning (Supplementary Fig. 1e, f). **d** The effects of the deletions are shown as the relative mRNA levels of Gsc and Smad7 used as a control of the TGF-beta signaling pathway. qRT-PCR analysis of Gsc mRNA expression in wild type (WT) or *Gsc* mutant clones and of Smad7 expression in activin A- (green) or SB431542- (gray) treated d3 cells. Gene expression level is normalized to WT samples, *n* = 3. Error bars represent s.e.m., *P* < 0.05, Mann–Whitney test using Prism 6 software (GraphPad Software). **e** Smad4 ChIP-qPCR data (*n* = 2) in ES cells showing that the 10 bp deletion in the GC-rich region abolished Smad4 interaction with this region without affecting Smad4 interaction with the Gsc +6 kb distal enhancer element. Error bar represents s.e.m

We first determined whether nodal-activated Smad2/3 and Smad4 in mouse ES cells interact with the GC-rich region of the *Gsc* promoter that binds to Smad4 in vitro^20^ (Fig. 1a, b). We analyzed nine human genome-wide ChIP-Seq data sets^9, 21–24^ that were available in the NCBI GEO database (Supplementary Fig. 1a, b). The proximal promoter (PP) in human *Gsc* contains one CAGAC motif, which is located between the FoxH1 site and the TSS, and is not conserved in mouse, whereas the PP in mouse *Gsc* contains three CAGA repeats, which lack a crucial bp of the CAGAC SBE motif (Supplementary Fig. 1c).

The analysis revealed binding of Smad2/3 and Smad4 to an area of the *Gsc* PP that encompasses the GC-rich region and the nearby FoxH1 binding site, but excludes the CAGA and CAGAC sites (Fig. 1a, b; Supplementary Fig. 1 a, b). Smad binding also occurred in putative distal enhancer (DE) elements that contain CAGAC sequences (Supplementary Fig. 1a, d). These results showed that in ES cells stimulated by TGF-β signals, Smad2/3, and Smad4 bind to a GC-rich region of the PP of *Gsc*.

To determine the functional relevance of the GC-rich region, we used CRIPSR/Cas9-mediated mutagenesis^25, 26^ to generate focal deletions in the *Gsc* PP in mouse ES cells. The homozygous deletion of a 155 bp segment including the FoxH1 binding motif and a large portion of the GC-rich region (Supplementary Fig. 1e) abolished the induction of *Gsc* by activin A (a ligand for nodal receptors) (Fig. 1c, d). Moreover, the homozygous deletion of a 10-bp segment within the GC-rich region (GCGCCGGGGC), which spared both the FoxH1 and CAGA sites, was sufficient to abolish the *Gsc* response (Fig. 1d). These deletions in the *Gsc* locus did not alter the response of a separate Smad target gene, the negative feedback regulator *Smad7*, in the mutant cell clones, indicating that the cells remain capable of activin signal transduction (Fig. 1d).

Additionally, we provide Smad4 ChIP-qPCR data in ES cells showing that the 10 bp deletion in the GC-rich region abolished Smad4 interaction with this region without affecting Smad4 interaction with the DE elements (Fig. 1e).

### Smad4 binding to GC sequences from the *Gsc* proximal promoter

**Fig. 2:**
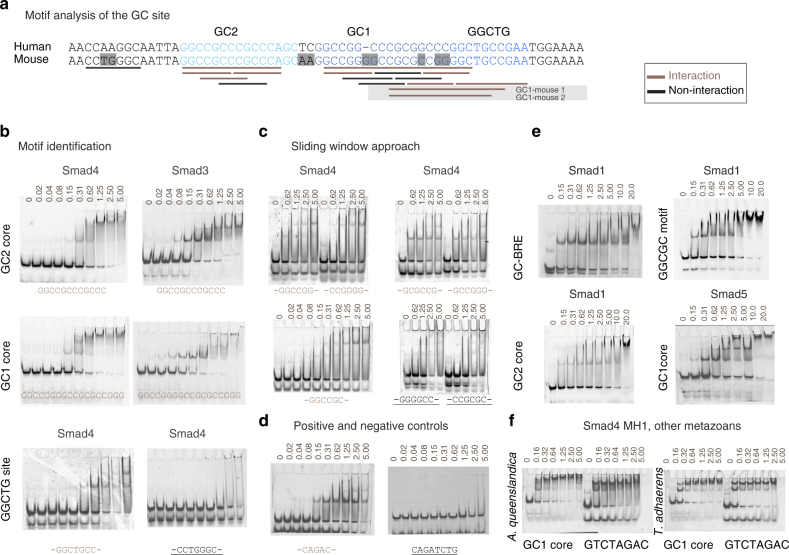
Identification of specific/unspecific GC binders using EMSA experiments. **a** Human and mouse comparison of the GC site (differences are highlighted in gray in the mouse sequence). Fragments of the GC site used for the EMSA assays are represented as bars under the sequences. Nanomolar affinity interacting fragments are shown as brown bars and sites that do not interact or that they do so in the micromolar range (considered unspecific) are in black. **b** Three regions of the GC site including the GC1 core, GC2 core, and the GGCTG site analyzed using EMSA assays. Experiments are performed using human Smad4 and Smad3 MH1 domains as indicated. Protein concentrations are shown on top of the EMSA (micromolar concentrations). Abbreviations for the DNA oligonucleotides referred to the binding motifs are shown and colored according to their interaction properties: brown for binders, black for non-binders. **c** Exhaustive analysis of the different motifs using EMSA experiments. The complete list of oligonucleotides is shown in Supplementary Table 1. Abbreviations for the DNA oligonucleotides referred to the binding motifs are shown and colored according to their interaction properties as in **b**. **d** Positive and negative binding controls using the CAGAC and CAGAT sites, respectively. **e** EMSAs showing the interaction of Smad1 and Smad5 to different DNA regions, showing affinities similar to those of Smad3 and Smad4. The SBE (GTCTAGAC) and GC-BRE sites (GGCGCC) were used as positive controls. Additional EMSAs are included in Supplementary Fig. 2a. **f** EMSAs corresponding to the Smad4 MH1 domains of sponge *Amphimedon queenslandica* and the placozoa *Trichoplax adhaerens* illustrating that binding to the GC sites is conserved in metazoans. The oligos used in these cases correspond to the GC1 core and to the canonical GTCT site (SBE)

In order to characterize the specific GC-rich motifs recognized by Smad proteins, we first designed two 30-bp dsDNA oligonucleotides (GC1 and GC2 segments, Fig. 2a), and measured the interactions by electrophoretic mobility shift assays (EMSA), using recombinant Smad MH1 domains and Cy5-labeled DNAs (Fig. 2b–d). To narrow down the motifs present in these regions, we applied a sliding window approach (6-bp windows sliding by 2-bp steps, within 20-bp duplexes, Supplementary Table 1) covering the GC1 and GC2 segments. The Smad4 MH1 domain-bound dsDNA oligonucleotides containing the GGCGC/G, GGCCGC/G, and GGCTG sequences (underlined in brown, Fig. 2a, b, and Supplementary Table 1) with an affinity in the nanomolar range, as determined by isothermal titration calorimetry (ITC) (Supplementary Fig. 2b). Smad4 bound the GGCGC oligonucleotide with a *K*_d_ of 160.3±0.2 nM. In parallel experiments, Smad4 bound a CAGAC oligonucleotide with *K*_d_ of 270.5±0.1 nM (Supplementary Fig. 2b).

The Smad3 MH1 domain bound the GC1 oligonucleotide with a *K*_d_ of ~100 nM, as estimated from EMSA (Fig. 2b). Smad1 and Smad8 MH1 domains also bind to these motifs efficiently with *K*_d_ values similar to those displayed for a GC-rich BRE and for the palindromic AGAC^15^ (Fig. 2e; Supplementary Fig. 2a). These values are comparable to dissociation constants of other transcription factors in and their cognate DNA motifs, which range from 10–100 nM^27–30^. Smad4 binding to these 5-bp GC-rich motifs is conserved through evolution, as determined with Smad4 MH1 domains from Placozoa and Porifera (Fig. 2f). Not all GC-rich motifs bound to Smad4 with high affinity. dsDNA oligonucleotide with sequences CCGCGC, CCTGGGC, and GGGGCC failed to bind the Smad4 MH1 domain in the nanomolar range (Fig. 2c), as did the FoxH1 site and a palindromic CAGA site sequence (Fig. 2d).

### Structural flexibility of the DNA-binding hairpin

**Fig. 3:**
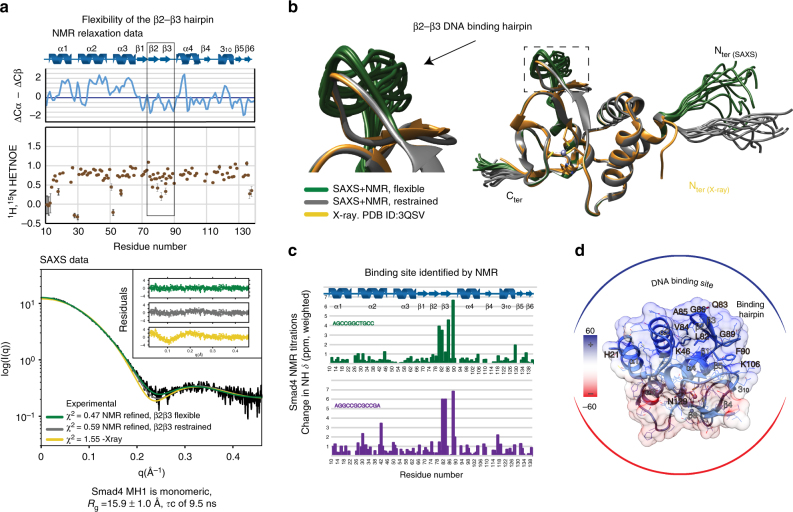
Structure and GC binding site of Smad4 MH1 domain in solution. **a** Heteronuclear NOEs values for all non-overlapped amides (shown as brown circles). Elements of secondary structure of Smad4 MH1 domain (10–140 residues) characterized using ^13^C chemical shift values (in blue) and NOE patterns are shown on top of the heteronuclear NOEs. Values of the β2–β3 DNA-binding hairpin are highlighted within a box, to indicate the flexibility of these residues in the absence of DNA. The SAXS scattering curves corresponding to a merged curve generated from data at several protein concentrations (0.5–4.2 mg mL^−1^) are shown below. *χ* ^2^ values of the different fittings are indicated. **b** Superimposition of the 10 best structures determined using the solution scattering curves SAXS refined with the NMR chemical shift values obtained in solution. **c** Identification of residues involved in DNA binding using NMR titrations. Chemical shift differences are shown as bars (green: AGCCGGCTGCC and blue: AGGCCGCGCCGA, respectively) using a 0.2 weighting of ^15^N with respect to ^1^H. The HSQC data displaying the chemical shifts induced upon binding are shown in Supplementary Fig. 3. **d** Residues affected upon DNA binding are displayed on the surface of the solution structure of Smad4 MH1 domain shown in **b**. The electrostatic charge distribution was calculated using PyMol

To investigate how Smad4 distinguishes between target and non-target GC-rich sequences, we first analyzed the properties of the MH1 domain devoid of DNA, using nuclear magnetic resonance (NMR) spectroscopy in solution. Flexibility and oligomerization properties of the MH1 domain were also analyzed by acquiring ^15^N NMR backbone relaxation data at two different protein concentrations. The ^15^N{^1^H} steady-state nuclear Overhauser effect (hNOE)^31^ values are homogeneous all along the sequence except for the β_2_–β_3_ pair, which forms the DNA-binding hairpin^3^. The β_2_–β_3_ hairpin is more flexible than the rest of the protein. (Fig. 3a) The analysis of the T1 and T2 experiments allowed us to calculate a correlation time *τ*_c_ of 9.5 ns, which is in agreement with a sample of 15 kDa tumbling as a monomer in solution.

These structural properties were confirmed by small-angle X-ray scattering (SAXS) profiles acquired at the European Synchrotron Radiation Facility (ESRF Grenoble, beamline BM29). The data suggest a maximum protein size of 56.5 ± 1.4 Å, (*D*_max_) consistent with a globular monomeric particle in solution with a radius of gyration (*R*_g_) of 15.9 ± 1.0 Å. (Fig. 3a) Remarkably, improving the fitting of the experimental data required refining of the X-ray model. The best results were obtained using the backbone chemical shifts converted to dihedral angles and allowing for some flexibility in the extended N-terminus region of the domain and in the β-hairpin involved in DNA binding. A set of the best 10 structures with the lowest *χ* ^2^ to the experimental SAXS profile^32^ superimposed to the X-ray structure (PDB: 3QSV) is shown in Fig. 3b.

Overall these results indicate that in the absence of DNA, the Smad4 MH1 domain is well structured and behaves as a monomer in solution. The fold is similar to that determined by X-ray crystallography of the Smad4 MH1 complex with DNA^13^, except that the DNA-binding hairpin presents conformational flexibility in the absence of DNA. We postulate that the flexibility of the hairpin facilitates access to DNA duplexes with slightly distinct topologies (as defined by different A/T or G/C contents), allowing the conserved hairpin to bind different DNA motifs. This hypothesis is supported by the NMR titration experiments performed with two different DNA motifs (Fig. 3c), which induce similar chemical shifts in the same residues located in and around the conserved β_2_–β_3_ hairpin (Fig. 3d). Notably, the sequence of this hairpin is highly conserved in all R-Smads and in Smad4 (Supplementary Fig. 2c)^33^, suggesting that the flexibility observed in Smad4 also occurs in R-Smads.

### High-affinity recognition of the GGCGC motif by Smad4

**Fig. 4:**
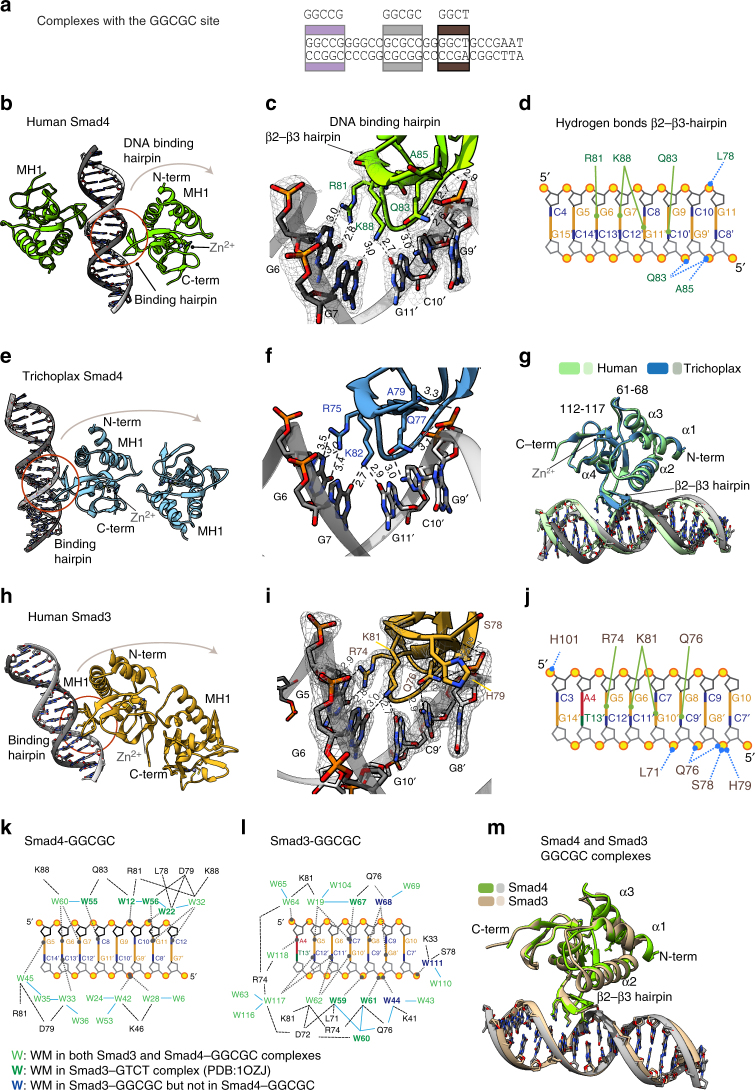
Smad4 and Smad3 MH1 domains bound to the GGCGC site. **a** Schematic representation of the GC1 site with the crystallized regions highlighted in gray. DNA sequences are shown in Supplementary Table 1. **b** Biological assembly of huSmad4 MH1–GGCGC complex (2.05 Å resolution). The hairpin binding site is circled. The bound Zn^2+^ and coordinating residues are shown. **c** A close view of the GGCGC recognition. Distances are shown in Å. The electron density corresponding to the binding region is contoured at 1σ level (2Fo-Fc). The stereoview representation of the complexes is shown as Supplementary Fig. 9. **d** Intermolecular contacts. Solid lines indicate hydrogen bonds between protein residues and DNA bases. Dashed lines indicate hydrogen bonds between residues and DNA phosphates. Bases are colored and labeled. **e** ASU of *Trichoplax adhaerens* Smad4 MH1 (shown in blue) with the GGCGC site (2.43 Å). **f** Specific intermolecular contacts of *ta*Smad4 MH1 with the GGCGC site. Distances shown in Å. **g** Superposition of the Human (green) and *Trichoplax* (blue and gray) Smad4 MH1 complexes with the same GGCGC DNA. Protein loops displaying minor structural differences are indicated (numbers correspond to the human sequence). **h** ASU of the huSmad3 MH1 in complex with the same GGCGC (2.05 Å). **i** Expanded view of the GGCGC site with bound Smad3. The electron density corresponding to the binding region is contoured at 1σ level (2Fo-Fc). **j** Intermolecular contacts for the huSmad3 MH1 in complex with the GGCGC site. **k** Summary of specific DNA–protein interactions mediated by water molecules for the huSmad4–GGCGC complex. Hydrogen bonds are represented by black dashed lines. Water molecules common to the huSmad3 GTCT complex (PDB:1OZJ) are shown in bold green, water molecules present in both Smad4 and Smad3 complexes are shown in green. Analyzed waters were selected as described in the Methods section (numbers correspond to those in the PDB files). **l** Summary of specific DNA–protein interactions mediated by water molecules for the Smad4–GGCGC complex, as in **k**. Waters only present in the Smad3 complex are shown in blue. **m** Superposition of the huSmad3 (tan) and huSmad4 (chartreuse) GGCGC complexes. Minor differences are detected at the α1–α2 and after α3. The DNA-binding site is nearly identical

Given that similar residues in the Smad4 MH1 domain interact with different DNAs, we set to identify which nucleotides directly contacted the protein. We applied high-throughput sparse matrix crystallization approaches using a set of 20 different duplexes, with varying DNA lengths and sequences, and obtained X-ray diffraction data of several complexes of Smad4 MH1 domain and these DNAs. The best diffracting crystals were obtained with DNAs of 16 and 18 bp. A complex with the 18 bp dsDNA oligonucleotide containing the GGCGCG sequence from the GC1 *Gsc* segment (Fig. 4a) yielded data at 2.05 Å (refer to Methods section and Table 1). The ASU (space group C222_1_) contains only one Smad4 MH1 monomer and a single DNA strand, while the second monomer and the complementary DNA strand in the biological assembly belong to the neighboring ASU (Fig. 4b).

**Table 1:** X-ray data collection and refinement statistics

	SMAD4-GGCGC 5MEY	SMAD4-GGCT 5MEZ	T_SMAD4-GGCGC 5NM9
*Data collection*			
Space group	C222_1_	P2_1_2_1_2_1_	P2_1_2_1_2_1_
Cell dimensions			
*a*, *b*, *c* (Å)	64.17, 79.00, 90.10	64.79,79.06,114.16	37.15, 76.98, 145.04
*α*, *β*, *γ* (°)	90, 90, 90	90, 90, 90	90, 90, 90
Resolution (Å)	50.00–2.05 (2.06–2.05)	30.00–2.98 (2.99–2.98)	30.00–2.43 (2.44–2.43)
*R*_meas_	6.7(118.3)*	9.4 (91.5)*	8.1(182.6)*
*I* /σ*I*	17.72(1.59)*	12.86 (1.68)*	12.32(0.80)*
Completeness (%)	99.9 (100)*	99.7 (100)*	98.7(100)*
Redundancy	6.2 (6.0)*	3.83 (4.06)*	4.4 (4.8)*
*Refinement*			
Resolution (Å)	29.70–2.05	29.05–2.98	29.43–2.43
No. reflections	14,709	12,239	16,190
*R*_work_/*R*_free_	0.222/0.238	0.217/0.252	0.221/0.251
No. atoms	1470	2534	2656
Protein	979	1927	1897
DNA	369	618	738
Zinc ions	1	2	2
Calcium ions	5	0	0
Water	92	7	19
B-factors			
Protein	48.35	75.36	73.32
DNA	50.76	96.29	105.01
Zinc ions	45.74	66.79	58.49
Calcium ions	107.54	0	0
Water	51.75	63.97	61.92
R.m.s. deviations			
Bond lengths (Å)	0.009	0.010	0.010
Bond angles (°)	1.00	1.05	1.02
	**SMAD3-GGCGC 5OD6**	**SMAD3-GGCT 5ODG**	**SMAD4-GGCCG 5MF0**
*Data collection*			
Space group	I4_1_	I4_1_	P4_3_
Cell dimensions			
*a*, *b*, *c* (Å)	105.20,105.20, 73.24	104.99, 104.99, 72.49	101.52,101.52,45.78
*α*, *β*, *γ* (°)	90, 90, 90	90, 90, 90	90, 90, 90
Resolution (Å)	30.00–2.00 (2.05–2.00)	30.00–2.12 (2.13–2.12)	30.00–3.03
*R*_meas_	9.0 (111.9)*	4.9 (66.30)*	11.6(98.7)*
*I* /σ*I*	7.51 (1.2)*	14.84 (1.70)*	12.06(1.76)*
Completeness (%)	99.5 (98.8)*	99.7 (98.4)*	99.8(98.8)*
Redundancy	2.90 (2.95)*	3.77 (3.64)*	4.9(5.1)*
*Refinement*			
Resolution (Å)	27.11–2.00	29.83–2.12	28.25–3.03
No. reflections	26,771	22,057	9298
*R*_work_ /*R*_free_	0.195/0.235	0.200/0.243	0.231/0.270
No. atoms	2863	2647	2608
Protein	2034	2026	1940
DNA	656	533	656
Zinc ions	2	2	2
Calcium ions	0	0	40
Water	167	81	4
B-factors			
Protein	55.01	62.2	62.52
DNA	81.93	84.12	104.88
Zinc ions	48.16	53.21	51.85
Calcium ions	0	0	0
Water	57.41	58.73	40.44
R.m.s. deviations			
Bond lengths (Å)	0.01	0.01	0.008
Bond angles (°)	1.00	1.06	0.97

* Values in parentheses are for highest-resolution shell

In the complex, the convex face of the DNA_-_binding hairpin dives into the concave major groove of the duplex DNA containing five base pairs (6-GGCGC-10/9′-GCGCC-13′). The main DNA-binding region of the protein comprises the loop following the β_1_ strand, and the β_2_–β_3_hairpin (residues 75–89 in Smad4). The Smad4 β_2_–β_3_ hairpin contains three residues Arg81, Gln83, and Lys88, which are strictly conserved in all R-Smad and Smad4 proteins and define specific interactions with three nucleotides in the canonical GTCT site previously investigated^14, 34^. In the complex with the 6-GGCGCG-11 site, these three residues also participate in a network of hydrogen bonds with the first four consecutive base pairs of the GGCGC motif.

The electron densities for these functionally important residues, the rest of the protein and DNA are well defined (Fig. 4c). Specifically, the guanidine group of Arg81 side chain coordinates tightly the G6 nucleotide. The orientation of Arg81 is further stabilized by the interaction of Asp79 side chain with the guanidine site of Arg81. In addition, Lys88 side chain is also well defined through interactions with both G7 and G11′, the latter being located in the complementary chain. The Gln83 side chain carbonyl interacts with C10′, while the amide part is in the proximity of the C9′ phosphate. The complex is further reinforced by a set of hydrogen bond interactions between the backbones of Leu78, Gln83, and Ala85, with G11, G9′, and C10′, covering the 6-GGCGCG-11 area (Fig. 4d) and by a network of well-ordered water molecules, which are tightly bound at the interface of the protein–DNA-binding site (Fig. 4k). Ten of the water molecules are closely coordinated by residues of the β2–β3 hairpin and six bases of the GGGCGCGC region. Additionally, five more water molecules strengthen the interactions of the N-terminal helix α2 with the phosphates of the DNA backbone.

These results indicate that the complex interface is highly complementary and that one MH1 protein covers a DNA-binding site of six base pairs. When compared to the structure of the Smad4–GTCT complex^13^, the proteins are nearly identical with minor differences detected in one of the loops and at the first helix (Cα RMSD of 0.39 Å for 113 aligned residues). Most of the differences concentrate at the DNA-binding interface.

We also solved the X-ray crystal structure of Smad4 MH1 in complex with the GGCCG sequence, which is present in both the GC1 and GC2 segments (Fig. 2e). The complex with GGCCG (data at 3.03 Å, Space group P4_3_) is similar to that with GGCGC, (Supplementary Fig. 4). The DNA sequence contains the GGCCG motif twice, and each of two bound MH1 monomers interacts slightly differently with this motif, with both binding sites in the biological unit displaying specific hydrogen bonds to either 5- or to 4-nucleotides, respectively. Data collection and statistics are shown in Table 1. These additional structures showed that Smad4 can recognize slightly different motifs (GGCGC and GGCCG) in the GC-rich region of *Gsc*.

### Smad4 recognition of the GGCGC motif across metazoans

We examined the recognition of the GGCGC site by the Smad4 MH1 domain of the Placozoa *Trichoplax*, metazoans whose bodies are composed by only four basic cell types and with one of the most divergent among the known Smad4 sequences relative to human Smad4 (Supplementary Fig. 2c). The crystal structure of the *Trichoplax* Smad4 MH1 domain bound to the GGCGC oligonucleotide reveals that this Smad4–DNA interaction is strictly conserved (Fig. 4e, f) (diffraction data obtained at 2.43 Å, space group P2_1_2_1_2_1,_ data collection, and statistics shown in Table 1). A superposition of the human Smad4 and *Trichoplax* Smad4 complexes shows that both complexes are very similar, and in particular the protein binding mode (Cα RMSD of 0.46 Å for 115 aligned residues). The few observed differences are concentrated in and around two loops that also contain most of the amino-acid sequence differences (Fig. 4g). Collectively, the results show that Smad4 proteins from highly divergent metazoan species interact with the GGCGC motif with similar binding affinity and similar structural contacts.

### Mode of Smad3 binding to the GGCGC motif

Next, we investigated the binding mode of the Smad3 MH1 domain with the GGCGC motif. Crystals of human Smad3 MH1 domain bound to a 16mer dsDNA oligonucleotide containing the GGCGCG motif yielded data at 2.05 Å resolution using synchrotron radiation (I4_1_ symmetry ID29, ESRF, Grenoble), which together with the Smad4 bound to the same site, are the highest-resolution structures determined for a DNA-bound Smad MH1 domain to date. The asymmetric unit (ASU) of the complex contains two Smad3 monomers and one dsDNA (Fig. 4h). Phases were obtained by molecular replacement using the crystal structure of the human Smad3 in complex with the GTCT motif, (PDB accession code: 1OZJ^34^). The electron density map obtained after refinement showed residues 10–135 of Smad3, and the dsDNA 16mer (data collection and statistics are shown in Table 1), including the presence of a bound Zn^2+^, coordinated by His126, Cys64, Cys109, and Cys121.

As in all other MH1 complexes, the three residues strictly conserved (Arg74 and Gln76 located in β_2_ and Lys81 in β_3_) are well defined in the structure and participate in a network of hydrogen bonds with the 5-GGCGCG-10 motif. The guanidine group of Arg74 is well ordered due to its tight coordination by the G5 nucleotide (fitting of the side chains to the density are shown in Fig. 4i). The Lys81 side chain is also well defined, through hydrogen bond interactions from the ɛ-amino group to the pair of nucleotides G6 and G10′ (in the complementary chain) and with several well-ordered water molecules in the proximity of the ɛ-amino group. The Gln76 side chain carbonyl interacts with N3 of C9′, and also with C7, G8, and G8′ mediated by the presence of several tightly bound water molecules (Fig. 4j, l). Various interactions occurred between Leu71, Gln76, Ser78, His79, and His101 backbone and the DNA phosphates. In addition, interactions from Lys33, Lys41, Leu71, Asp72, Arg74, Gln76, Ser78, and Lys81 to the DNA via bound water molecules, further stabilize the complex. In the past, it has been suggested that binding of the Smad3 MH1 domain to GC-containing motifs might be unspecific through contacts made from lysine and arginine residues present in helix α2 with the DNA^34^. However, among the many hydrogen bonds detected from the protein to DNA, only two of them involved two residues located in this helix and were facilitated via water molecules. In fact, Smad3 and Smad4 GGCGC complexes are structurally very similar with a Cα RMSD of 0.44 Å for 105 aligned residues and both complexes interact directly with the DNA using the β_2_–β_3_ hairpin (Fig. 4m).

### Smad3 and Smad4 interactions with the GGCT motif

**Fig. 5:**
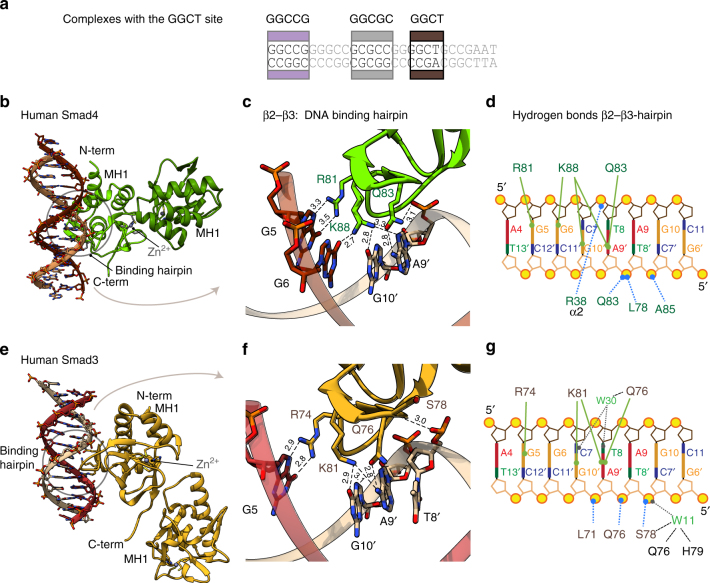
Complex structures of the Smad4 and Smad3 MH1 domains with the GGCT site. **a** Schematic representation of the GC1 site. The GGCT DNA used for crystallization was TGCAGGCTAGCCTGCA and its location in the GC1 site is highlighted in dark brown. **b** Asymmetric unit of Smad4 complex with the GGCT motif. The ASU contains two Smad4 MH1 domains and one dsDNA. **c** Smad4 complex with the GGCT motif, as in Fig. 4c. Lys88 is displayed with two side chain orientations, as present in the different molecules of the ASU. **d** Intermolecular contacts for Smad3 complex with the GGCT motif, as in Fig. 4d. **e** Smad3 complex with the GGCT motif. The asymmetric unit of the complex contains two Smad3 MH1 domains and one dsDNA. **f** Smad3 complex with the GGCT motif, as in Fig. 4c. **g** Intermolecular contacts for Smad4 complex with the GGCT motif, as in Fig. 4d

The GC-rich region of *Gsc* contains a conserved GGCTG sequence, which resembles the GTCT-AGAC SBE (Fig. 5a). This GGCTG motif bound Smad3 and Smad4 MH1 domains with high affinity in the EMSA experiments. We determined how the T to G change modified the recognition of the motif by Smad3 and Smad4 using a palindromic version of the motif similar to the canonical SBE. The X-ray crystal structure of the Smad4 MH1 domain in complex with a GGCT-AGCC oligonucleotide was determined at 2.98 Å resolution (space group P2_1_2_1_2_1_). The ASU contains two Smad4 MH1 monomers and one dsDNA molecule (Fig. 5b). Contacts were detected from Arg81 to G5 in one DNA strand and from Gln83 to A9′ in the complementary DNA strand. Lys88 is bound by G10′ in one of the monomers, as in the previously described complex with the canonical GTCT complex (PDB: 3QSV). Interestingly, in the second monomer, the density of Lys88 suggests that both G6 and G10′ nucleotides are contributing to the interaction with the GGCT motif, similar to what we observed in the GGCGC complex (both conformations are shown in Fig. 5c, d).

The Smad3 MH1 domain complex with the GGCT oligonucleotide was refined at 2.12 Å resolution (space group I4_1_) (Fig. 5e). Most of the hydrogen bonds with the GGCT site are very similar to those observed with Smad4 (Fig. 5f, g). The main difference is that in the Smad3 complex no hydrogen bonds are observed from the ɛ-amino group of Lys81 to the G6 nucleotide. In this complex, we also detected several tightly bound water molecules at the protein–DNA interface that contribute to the stabilization of the interactions (Fig. 5g).

### Comparative analysis of Smad binding to different motifs

**Fig. 6:**
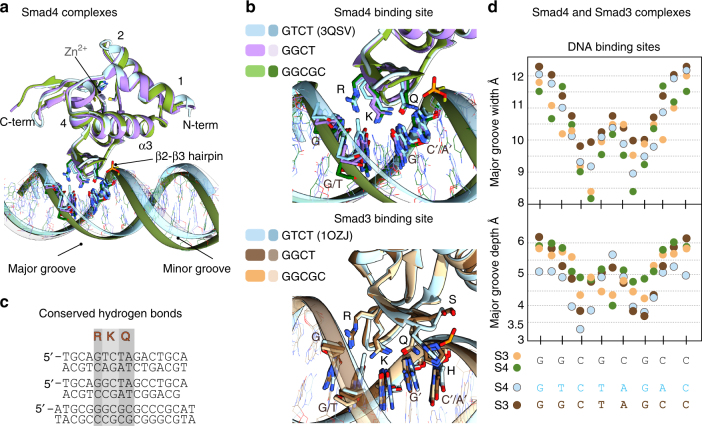
Consensus binding site for GC motifs and DNA shape comparison. **a** Superposition of the human Smad4 complexes with the GGCGC site (shown in green) and the GGCT site (in violet) to the GTCT structure (sky blue) previously determined (PDB: 3QSV). **b** Superposition of Smad4 (Top) and Smad3 (bottom) binding sites. Nucleotides that interact with the protein are highlighted. The Smad4 complexes are colored as in **a**. Smad3 complexes are displayed in tan (GGCGC site), brown (GGCT), and light blue GTCT (structure previously determined PDB:1OZJ). **c** Structural alignment of the DNA-binding sites observed for the six complexes, with the contacts to the three conserved residues R, K, and Q highlighted. **d** DNA shape comparison for different complexes. Major groove width (top) and depth (bottom) were calculated using Curves+ for Smad3 MH1–GGCGC complex (tan), Smad3 MH1–GGCT (brown), Smad4 MH1–GGCGC complex (green), and Smad4 MH1 (light blue) bound to the GTCT site (PDB: 3QSV). The analyzed sequences are indicated

We analyzed the superposition of the Smad4 MH1 domain bound to different DNAs (Fig. 6a) with Cα root mean square deviations (RMSD) of 0.47 Å and of 0.45 Å for the GTCT and GGCT, respectively, for 114 aligned residues. Similar RMSD values were obtained when we compared the Smad3 complexes. Both GGCGC and GTCT structural comparisons reveal differences in the DNA-binding mode, including the number of direct hydrogen bonds to the bases, and in the topology of the DNA structures, (Fig. 6b), although the same protein residues are involved in the interactions (Fig. 6c). The complexes of Smad4 or Smad3 MH1 domain with the ds-GGCGC motif show a narrower but deeper major groove and a wider and less pronounced minor groove (average width of 10.0 and 6.5 Å, respectively for the Smad4 complex) compared to this domain bound to GTCT/AGAC (3QSV) (10.6 and 5.9 Å, respectively). The conformation of the Smad-bound GGCT motif is intermediate between that of GTCT and that of GGCGC. Similar values were measured for Smad3 complexes. The similarities and differences of both topologies were quantified using Curves+^33^ (Fig. 6d). Similar topologies of GC-rich DNAs have been previously reported in other cases including the binding of several consecutive Zn-finger domains to GC motifs^35^. These differences in the DNA shape and flexibility provide a good match between the convex face of the DNA-binding Smad hairpin and the concave and narrow major groove of the GGCGC site, allowing the side chains of Arg81 and Lys88 to establish a well-defined set of hydrogen bonds with G nucleotides.

### A consensus GC-rich Smad binding element

The six MH1–DNA complex structures reveal common features of how Smad3 and Smad4 recognize GC motifs in a specific manner. They also illustrate why not all GC motifs can be Smad binding targets. Efficient interactions with GC sites occur only if a G nucleotide is located deep in the major grove, and establishes hydrogen bonds with the guanidinium group of Arg81. This interaction facilitates a complementary surface contact between the Smad DNA-binding hairpin and the major groove of the DNA. The ε-amino group of Lys88 also displays hydrogen bonds with two nucleotides in positions+1 and +2 (also guanines) with respect to the first G. Due to the extended side chain of the lysine, the guanines can be located at either side of the DNA duplex. These conserved sets of contacts define the first three nucleotides in the preferred motifs. Gln83 is more versatile and can form hydrogen bonds using its bi-functional side chain. Indeed, Gln83 interacts with GC in GGCGC (or CG in GGCCG) and with an adenosine in GTCT and GGCT.

Additional contacts are observed with the DNA backbone to define an area of interactions with up to 6 bp. The pattern of contacts and the binding mode elucidated here for Smad3 and Smad4 with the GGCGC motif are slightly different to that recently described for Smad5 bound to the palindromic GGCGCC site^15^. In the Smad5 complex, perhaps favored by the short palindromic nature of the motif, the binding mode is defined by two MH1 domains bound to the six nucleotides DNA site, with each domain binding only three contiguous nucleotides^15^. However, in the Smad3 and Smad4 complexes, we find that five nucleotides are covered by a single MH1 domain through direct interactions, and up to six nucleotides when we include hydrogen bonds with the DNA backbone. The comparison of the different hydrogen bonds detected in the Smad3 and Smad4 GGCGC complexes to those described for Smad5 bound to the GGCGCC site is included as (Supplementary Fig. 5).

The observed pattern of interactions defines a consensus GC-rich binding site of five nucleotides, the GGC(GC)|(CG) motif, that binds with high affinity to MH1 domains of Smads 1, 2, 3, 4, and 8. The 5 bp GGC(GC)|(CG) motif covers five bases of the GGC palindrome and a GCCG motif, which were previously identified as BREs^36^. We propose the term 5GC SBE to designate this general SBE.

### 5GC Smad binding elements are clustered

**Fig. 7:**
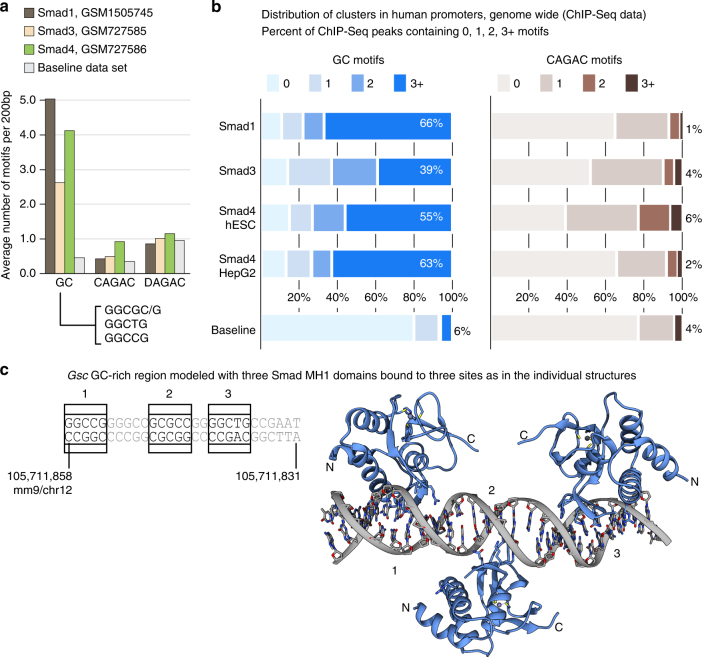
5GC motifs are enriched in nodal/TGF- β and in BMP-stimulated cells CREs. **a** Bar representation showing the average number of GC (GGCGC/G, GGCTG, and GGCCG), CAGAC, and DAGAC motifs found in 200 bp-regions of Smad1, Smad3, and Smad4 ChIP-Seq data up to 1000 bp from the TSS (shown in brown, beige, and green, respectively) or from the baseline data set (gray). AGAC motifs preceded by nucleotides different from C are included under the DAGAC name. **b** Stacked bar plots showing the number of motifs per 200 bp region (as %) found in Smad1, Smad3, and in Smad4-bound ChIP-Seq data, considering the promoter regions as above. Smad4 data corresponding to HepG2 cell lines are shown for comparison of the distribution of motifs to ES. Regions are classified as 0, 1, 2, 3, or more sites. Left: GGCGC/G, GGCCG, GGCTG sites together vs. baseline, colored in blue. Right: CAGAC motifs vs. baseline, colored in brown. **c** Model of the GC region with the three Smad DNA structures determined. Although based on the sequence, there is an additional potential binding site in this region, only three binding sites are available. Once the first domain is bound (in one of the places), there are only two more sites accessible. With this arrangement, there is no direct contacts between the MH1 domains

We investigated the presence of the 5GC SBE motifs in Smad1, Smad3, and Smad4 in human ES cells and derived endoderm ChIP-Seq peaks (GSM1505745, GSM727586, and GSM727585 data sets), focusing on CREs genome-wide. For a given transcription start site (TSS), we selected 200 bp DNA regions centered at the ChIP-Seq peak, within regions covering to 1000 bp upstream of the TSS. As baseline, we selected 500 regions of 200 bp length at random coordinates. We found the 5GC SBE motifs GGCGC/G, GGCCG, and the GGCTG motif to be 10-fold enriched in Smad1 ChIP peaks compared to the baseline, whereas the CAGAC motif is at the baseline levels (Fig. 7a). The analysis revealed that the 5GC SBEs present in hESC Smad ChIP peaks (in promoters) frequently appear in clusters, as it occurs with *Gsc*, with 66% of the regions including three or more 5GC motifs in Smad1. This is in contrast to CAGAC motifs, which are present as clusters in only 1% of the Smad1 ChIP peaks (Fig. 7b). For the Smad4 ChIP peaks, the 5GC is enriched eight-fold and CAGAC motifs are enriched three-fold compared to the baseline (Fig. 7a). In the case of Smad3 ChIP peaks, the 5GC is enriched five-fold and CAGAC motifs are enriched two-fold compared to the baseline (Fig. 7a). The 4-bp AGAC motif is not enriched in ChIP-Seq regions (1.17 motifs per 200 bp region in ChIP peaks vs. 1.04 in controls, excluding CAGAC motifs). Similar values of cluster enrichment were observed in Smad3 (39% of regions with three or more 5GC motifs vs. 4% of CAGAC), and Smad4 (55% 5GC vs. 6% CAGAC). We also analyzed the 5GC clustering in a Smad4 ChIP-Seq in human hepatocarcinoma (HepG2) cells (Encode ENCFF484WVM data set). In this data set, we found similar results with respect to the hESC cluster enrichment, with 63% of the peaks showing three or more 5CG motifs vs. 2% of CAGAC clusters. Finally, a quarter of the promoter regions studied in the three Smad ChIP-seq data sets contain clusters of three or more 5GC SBEs accompanied with CAGAC motifs. Some examples of the identified clusters in TGF-β/nodal and BMP target genes are shown in Supplementary Figs. 6 and 7, respectively. In addition, to explore the 5GC cluster enrichment outside of promoter regions, we also performed these analyses using all Smad4 ChIP-Seq peaks genome-wide (Supplementary Fig. 8).

As revealed by the structures with the 5GC motifs, the fact that specific contacts in the complexes involve nucleotides present in both DNA strands provides an additional plasticity to the interactions but also adds complexity for the identification of these motifs in ChIP-Seq data analysis genome wide, explaining why the GC motifs have remained elusive in these analyses.

## Discussion

The Smad MH1 domain is highly conserved in distantly related metazoans, with no amino-acid sequence differences in the DNA-binding β-hairpin (with the exception of *Drosophila* Smad2). The structures determined here reveal that the β-hairpin is flexible and adaptable to bind with high-affinity several variants of the consensus sequence GGC(GC)|(CG), which we refer to as 5GC SBE. The structures also define the contacts of the Smad3 and Smad4 MH1 domains with specific DNA bases and phosphate groups.

Given the sequence identify of the β-hairpin and the overall high conservation of the MH1 domain in all R-Smad proteins and Smad4, it is not surprising that the various 5GC SBE motifs similarly bind to Smads 2 and 3, which operate in the TGF-β/nodal signaling, Smads 1, 5, and 8, which operate in BMP signaling, and Smad4, which is shared in all of these pathways. Binding of Smads 3 and 4 to the 5GC motifs is of similar or higher affinity than the binding of these proteins to the CAGAC motif GTCG (and to its variant GGCT), which has long been considered as a TGF-β responsive SBE. Smads 3 and 4 also bind to the motif GGCGCG, which is similar to the palindromic GGCGCC sequence previously identified as a BRE for BMP-activated Smad1^16^.

The present results challenge long-standing perceptions that a dichotomy exists between the intrinsic preference of different Smad proteins for different SBEs. The concept that Smad2/3 and Smad4 primarily bind CAGAC SBEs, whereas Smads 1, 5, and 8 prefer GC-rich motifs seem too restrictive in light of the evidences. We propose that all R-Smad and Smad4 proteins recognize G(T/G)CTG motifs of the classical CAGAC SBE as well as GGC(GC)|(CG) motifs of the 5GC SBE. Analysis of ChIP-Seq data sets corresponding to CREs indicates that Smad responsive regions frequently contain both 5GC and GACAG SBEs, with a higher abundance of 5GC SBEs, which frequently occur as clusters. Additional biochemical and functional studies would need to be done in the future to validate the genome-wide findings and to narrow down the specific regions recognized by Smad proteins case by case.

Smad proteins do not bind to just any 5-bp GC-rich motif, and our structures of Smad–DNA complexes illuminate the basis for the selectivity of these interactions. These insights also suggest how three MH1 domains in a trimeric Smad complex may bind to DNA at once. The clustering of multiple SBEs in gene promoter regions may facilitate the cooperative binding of three Smad molecules in the same complex to a target promoter. This is illustrated by a model of three Smad MH1 domains binding to 5GC SBEs in the *Gsc* PP (Fig. 7c).

The regulation of distinct gene sets by TGF-β and BMP signals is dictated by the differential binding of Smad2/3 and Smad1/5 to master lineage-determining transcription factors, such as FoxH1 in the context of mesendoderm progenitors, and other transcription factors in other cellular contexts^1^. LDTFs bound to cognate sites throughout the genome recruit activated Smad complexes to these loci, thus dictating where Smads may bind to DNA if appropriate motifs are regionally available in these loci. In this model, the target gene specificity of TGF-β vs. BMP-activated Smads is primarily dictated by the selective affinity of certain LDTFs for Smad2/3 or Smad1/5. The flexibility of the DNA-binding β-hairpin to recognize different variants of the 5GC and CAGAC SBEs, as shown here, combined with the occurrence of these SBEs in clusters, facilitates the binding of the LDTF-recruited Smad trimer to DNA for the assembly of functional transcriptional complex. This capacity also endows Smad transcriptional complexes with a level of adaptability that may have contributed to establish TGF-β signaling as one of the most versatile and highly conserved signaling pathways in metazoans. Our findings suggest a molecular basis for the functional versatility of Smad complexes in different biological contexts.

## Methods

### Protein expression and purification

The plasmid (Addgene #14959) encoding the human Smad4 protein was used as the template for cloning different constructs of the MH1 domains to optimize the yield and homogeneity of the recombinant proteins. The best results were obtained with the Pro10-Gly140 fragment of Smad4, which was expressed as a N-terminal His-tagged fusion protein followed by a TEV or 3C cleavage sites. The MH1 domain is very stable in solution, showing a melting temperature of 66.2 ± 0.1 °C determined by differential scanning calorimetry and well folded according to the NMR data. The 1D and 2D NMR data of this recombinant domain show a well-dispersed pattern of chemical shifts indicative of a folded sample (^1^H, ^15^N-Heteronuclear 2D Single Quantum Coherence, and Transverse Relaxation-Optimized SpectroscopY, HSQC-TROSY).

Two divergent Smad4 MH1 domains, (*Amphimedon queenslandica*, (Porifera) NCBI Reference Sequence: XP_003388571, Thr52-Thr192; and *T. adhaerens* (Placozoa), GenBank: EDV21247, Met1-Ser151) as well as the Hu-Smad3 (NCBI Reference Sequence: NP_005893, isoform 1, Met-Pro141), the Smad8/9 domain (Uniprot: O15198-1, Thr14-Pro144), Smad1 (Q15797-1, Thr10-Arg142), Smad5 (Q99717-1, Thr11-Arg 143) were cloned using a synthesized DNA template with optimized codons for bacterial expression (Thermo Fisher Scientific). All clones were confirmed by DNA sequencing.

All protein constructs were expressed in *Escherichia coli* BL21 (DE3) and purified following standard procedures. Unlabeled and labeled samples were prepared using LB and minimal media (M9) cultures, respectively. D_2_O (99.95%, Silantes), ^15^NH_4_Cl, and/or d-[^13^C] glucose (Cambridge isotopes) were used as sole hydrogen, nitrogen, and carbon sources, respectively, to prepare the labeled samples^37^. Proteins were expressed fused to a N-terminal His-tag followed by a TEV protease cleavage site.

Cells were cultured at 37 °C to reach an OD_600_ of 0.6. After induction with IPTG (final concentration of 0.4 mM) and overnight expression at 20 °C, bacterial cultures were centrifuged and cells were lysed using an EmulsiFlex-C5 (Avestin) in the presence of lysozyme. The soluble supernatants were purified by nickel-affinity chromatography (HiTrap Chelating HP column, GE Healthcare Life Science) using a NGC Quest 10 Plus Chromatography System (BIO-RAD). Eluted proteins were digested with TEV or 3C proteases (at 4 °C or room temperature, respectively), and further purified by ion exchange chromatography using a HiTrap SP HP (GE Healthcare), and size-exclusion chromatography on a HiLoadTM Superdex 75 16/60 prepgrade columns (GE Healthcare) equilibrated in 20 mM sodium phosphate (pH 6.3), 150 mM NaCl, and 2 mM TCEP (Melford, UK). For crystallography (Smad4 MH1 domains), the last step of purification was performed using 20 mM Tris buffer, (pH 7.2), 80 mM NaCl, and 2 mM TCEP. All clones were confirmed by DNA sequencing and the purified proteins were verified by MALDI-TOF mass spectrometry.

### Duplex DNAs

Duplex DNAs were annealed using complementary single-strand HPLC purified DNAs. DNAs were mixed at equimolar concentrations (1 mM), heated at 90 °C for 3 min and allowed to cool down to room temperature during 2 h. DNAs were purchased (at Biomers and/or at Metabion, Germany).

### Electrophoretic mobility shifts assay

Binding reactions were carried out for 30 min at room temperature in 10 μL of binding buffer (20 mM Tris pH 7.2, 80 mM NaCl, 2 mM DTT). A fixed concentration of 5′-end Cy5-labeled duplex DNA (7.5 nM) was incubated with increasing amounts of Smad4 MH1 domain. Electrophoresis was performed in non-denaturing 12.0% (19:1) polyacrylamide gels. The gels run for 1 h in 1× TG buffer (25 mM Tris, pH 8.4, 192 mM Glycine) at 90 V at 20 °C. The gels were exposed to a Typhoon imager (GE Healthcare) using a wavelength of 678/694 nm (excitation/emission maximum) for the Cy5 fluorophore.

### NMR chemical shift assignment and perturbation experiments

NMR data were recorded at 298 K on a Bruker Avance III 600-MHz spectrometer equipped with a quadruple (^1^H, ^13^C, ^15^N, ^31^P) resonance cryogenic probe head and a z-pulse field gradient unit at 298 K. Backbone ^1^H, ^13^C, and ^15^N resonance assignments were obtained by analyzing the TROSY versions of 3D HNCACB and HN(CO)CACB experiment pair using (^1^H/^2^H, ^13^C, ^15^N samples)^38^. To optimize the quality of the triple resonance backbone spectra, all experiments were acquired as Band-Selective Excitation Short-Transient-type experiments (BEST) with TROSY and non-uniform sampling (NUS), using two different buffer conditions (pH 7.5 and 5.5) to minimize the number of overlapped amides^38, 39^. This strategy allowed us to unambiguously assign 116 of the 125 possible amides (131 residues, 6 of them prolines). The comparison of the Smad4 MH1 (Cα and Cβ) chemical shifts to reference values, as well as the ^15^N edited-NOESY data, corroborated the presence of bound Zn^2+^ and of four helices and six strands, characteristic of the MH1 fold. The strands are ordered as three anti-parallel pairs: β_1_β_5_, β_2_β_3_, and β_4_β_6_. The presence of many long-range interactions confirmed that, the structure of the MH1 domain is well defined in solution, in the absence of DNA. Chemical shifts have been deposited in the BMRB (entry 26945). For the titration experiments, NUS acquisition strategy was also used to reduce experimental time and increase resolution. Protein samples (500 μM for backbone and 15N-NOESY experiments and 100 μM for titrations) were equilibrated in a buffer containing 20 mM sodium phosphate and 100 mM NaCl. All samples were supplemented with 10% D_2_O and pH adjusted to 6.3. Spectra were acquired using ^15^N-labeled protein samples at the indicated concentrations with progressively increasing amounts of the unlabeled DNA. Chemical shift perturbation analyses were performed with a 0.2 weighting of ^15^N with respect to ^1^H. NMRPipe^40^ and MddNMR^39^ were used for spectra processing and spectra assignment and analysis was performed with CARA^41^.

### Genome-editing with CRISPR/Cas9

sgRNAs targeting genomic regions of interest were designed using CRISPR Design Tool (http://crispr.mit.edu/)^42^ and synthesized by IDT, Inc. Single cells were sorted onto irradiated MEF feeder for increased viability through FACS 72 h post transfection. Mutant clones were first screened through aberrant melting temperature of qPCR products, then verified by PCR, TA cloning, Sanger sequencing, and CRISPResso analysis individually. The mESCs used for this experiment are diploid.

### Chromatin immunoprecipitation

mESCs E14Tg2a.IV (RRID: MMRRC_015890-UCD) were maintained on gelatin- (0.1%, Millipore, ES-006-B) coated plates with LIF-supplemented medium at 37 °C with 5% CO_2_. Basic ES cell medium included 80% knockout DMEM (Life Technologies, 10829-018), About 15% fetal bovine serum (HyClone, SH30071), 50 U Penicillin and 50 μg/mL Streptomycin (Cellgro, 30-001-CI), 1% non-essential amino acids (Life Technologies, 11140-050), 1% l-glutamine (Life Technologies, 25030081), 100 μM β-mercaptoethanol (Sigma-Aldrich, M6250), 1000 U/mL mLIF (Gemini Bio-Products, 400–495). EB formation and differentiation were carried out as described by the supplier (ATCC).

Mouse EBs were treated with recombinant human activin A (R&D Systems, 338-AC 50 ng/mL), fixed and quenched, sonicated to average fragment size of 250 bp in 1% SDS lysis buffer, and incubated with 60 μl Dynabeads protein G conjugated with 3–5 ug of indicated antibodies (SC-7966X, Santa Cruz Biotechnology). About 2% pre-cleared chromatin prior to primary antibody addition was kept as input DNA. Magnetic beads were washed, chromatin was eluted, and reverse crosslinked ChIP DNA was dissolved in 10 mM Tris pH 8.0 buffer for further analysis.

ChIP-Seq DNA samples were quantified and quality assessed by Ribogreen and Agilent Bioanalyzer. DNA fragments range from 200 to 600 bp were selected constructed for ChIP-Seq library with TruSeq ChIP Sample Prep Kit (Illumina) according to manufacturer’s instructions. Multiplexed sequencing libraries were run on a Hiseq2500 platform. Sequencing reads in FASTQ format were quality assessed by FastQC v0.11.3 for sequencing integrity and completeness. FASTQ reads were mapped to mouse genome mm9 (NCBI build 37, Jul/2007) with Bowtie2 with default filtering criteria. Resulted SAM files were converted to BAM files though Samtools 0.1.19. BAM files were sorted and indexed with Samtools. BAM files were converted to TDF file by IGV Tools 2.3.32 using the command “igvtools count -z 5 -w 25 -e 250”, specifying the coverage window size to be 25 bp and average fragment size of 250 bp. Additional information is described in the GSM2746361 entry.

### NMR relaxation measurements

T1 and T2 relaxation measurements were acquired using standard pulse sequences^43^. Peak integration values were fitted to a two-parameter function (Eq. 1), where *I*_0_ and *I(t)* are the peak intensities at times 0 and *t*, respectively. The optimum value of the *I*_0_ and the *T*_1,2_ parameters are determined using the Levenberg–Marquardt optimization algorithm:

1 $$I\left( t \right) = I_0e^{\left( {\frac{{ - t}}{{T_{1,2}}}} \right)}$$

The rotational correlation time of the domains was calculated with the Eq. 2, using the approximation of slow molecular motion *τ*_c_ larger than 0.5 ns and assuming that only J(0) and J(ωN) spectral density terms contribute to the overall value. νN is the ^15^N resonance frequency:

2 $$\left( {60.08 \times 10^6{\mathrm{Hz}}} \right)\tau _{\rm{c}} \approx \frac{1}{{4\pi \nu _N}}\sqrt {\left( {6\frac{{T_1}}{{T_2}} - 7} \right)}$$

Heteronuclear NOE experiments were acquired in an interleaved manner (the reference and the presaturated HSQC spectra). Steady-state values of ^1^H-^15^N NOEs resulted from the ratios of the peak intensities measured in the reference (*I*_0_) and the presaturated (*I*_S_) spectra during the relaxation delay, as described. Background noise levels *σ*_S_ and *σ*_0_ were measured and used to determine the NOE standard deviation:

3 $$\frac{{\sigma _{{\rm{NOE}}}}}{{{\rm{NOE}}}} = \left( {\left( {\frac{{\sigma _{I_{\rm{S}}}}}{{I_{\rm{S}}}}} \right)^2 + \left( {\frac{{\sigma _{I_0}}}{{I_0}}} \right)^2} \right)^{\frac{1}{2}}$$

### SAXS data collection, analysis, and modeling

SAXS data were collected on samples of human Smad4 MH1 at protein concentrations ranging from 0.5 to 4.2 mg mL^−1^, in 20 mM Tris buffer, pH 7.2, 150 mM NaCl. Data were acquired at Beamline 29 (BM29) at the European Synchrotron Radiation Facility (ESRF; Grenoble, France). Protein samples were centrifuged for 10 min at 10.000×*g* prior to data acquisition. Experiments on BM29 were collected at 12.5 keV and data were recorded on a Pilatus 1 M detector, at 10 °C. We collected 10 frames for each of the samples (1 s exposure).

Image conversion to the 1D profile, data reduction, scaling, and buffer subtraction were done by the software pipeline available at the BM29 beamline. Further processing was done by the ATSAS software suite^44^. Guinier plot calculation, for radius of gyration estimation, was performed by PRIMUS, included in the ATSAS suite, using low *q* regions (*q*_max_ × *R*_g_ < 1.3).

Modeling with SAXS and NMR data were done with XPLOR-NIH^45^, using the SAXS 1D profile^46^ and the backbone chemical shifts of Smad4 MH1. The chemical shifts were converted to backbone, phi, and psi, dihedral angles using TALOSN^47^. Starting from the crystal structure (PDB: 3QSV), DNA and water molecules were removed and secondary structure elements were restrained with an RMSD potential, implemented in XPLOR-NIH. A simulated annealing procedure was applied using the SAXS and dihedral angles as restraints. Comparison between experimental and fitted SAXS profiles was done using CRYSOL^32^. The 10 structures, with the lowest *χ* ^2^ to the experimental SAXS profile, were selected for further analysis.

Calculation of the *χ* ^2^ metric, for *N* data points, is given by:

4 $$\chi ^2 = \frac{1}{N}\mathop {\sum }\limits_{i = 1}^N \frac{{\left[ {I^{{\rm{calc}}}\left( {q_i} \right) - I^{{\rm{obs}}}\left( {q_i} \right)} \right]^2}}{{\sigma _i}}$$

### X-ray

High-throughput crystallization screening and optimization experiments were performed at the HTX facility of the EMBL Grenoble Outstation^48^. The human and *Trichoplax* Smad4 as well as the human Smad3 MH1 domains were concentrated to 5 mg mL^−1^ prior to the addition of the annealed DNAs (Metabion) dissolved in 20 mM Tris pH 7, 10 mM NaCl. The final protein DNA molar ratio was 1:1. Screenings and optimizations were prepared by mixing 100 nL of the complex solution and 100 nL reservoir solution in 96-well plates. Crystals of the complexes were grown by sitting-drop vapor diffusion at 4 °C. Crystals were obtained with several DNAs and conditions and were reproducible. Several data sets were acquired for the best diffracting crystals and analyzed. Final conditions for the three best diffracting complexes were optimized as follows:

Human_Smad4 GGCGC complex: the crystallization condition was 17% PEG6000, 0.2 M sodium chloride, and 0.1 M sodium acetate at pH 5.0 in the crystallization buffer. Crystals were cryoprotected in mother liquid supplemented with 20% PEG MME 500. The purification buffer was supplemented with 20 mM calcium chloride.

Human_Smad4 GGCCG complex: 16% PEG MME 2000 and 0.1 M sodium acetate pH 5.0. Crystals were cryoprotected in mother liquid supplemented with 20% glycerol.

Trichoplax_Smad4 GGCGC complex: 4.8% PEG4k, 0.1 M sodium acetate at pH 4.6. Crystals were cryoprotected in mother liquid supplemented with 20% PEG MME 550.

Human_Smad4 GGCT complex: 24% PEG3350 and 0.2 M sodium chloride. Crystals were cryoprotected in mother liquor supplemented with 18% PEG MME 550.

Human_Smad3 GGCGC complex: the crystallization condition was 0.2 M lithium acetate, 20% PEG3350. Crystals were cryoprotected in mother liquid supplemented with 20% 550 MME.

Human_Smad3 GGCT complex: 0.02 M sodium potassium phosphate, 0.1 M BisTris propane pH 6.5, 20% PEG3350 cryoprotected with 20% 550 MME.

Crystals were frozen in liquid nitrogen. Diffraction data were recorded at the ESRF on the beamlines ID23-2 (for Hu_Smad4-GGCGC and Hu_Smad4-GGCCG), ID23-1 (for T_Smad4-GGCGC), or ID30A-3 (for Hu_Smad4-GGCT) and ID29 (for Hu_Smad3-GGCGC and GGCT complexes). The data were processed with XDS^49^ and scaled and merged with XSCALE^50^. Initial phases were obtained by molecular replacement using PHASER^51^ from the CCP4 suite (search model PDB code: 3QSV). REFMAC^52^, PHENIX^53^, and BUSTER^54^ were employed for the refinement and COOT^55^ for the manual improvement of the models. Water molecules bound at the DNA–protein interface were selected when they participate in at least three hydrogen bonds (cutoff distance of 3.5 Å).

### Ramachandran statistics

The human Smad4–GGCGC complex has 99.2 and 0.8% of the residues in the preferred and allowed regions of the Ramachandran plot, 96.7 and 3.3% for the *Trichoplax* Smad4-GGCG, 96.7 and 3.3% for the GGCT complex, 98 and 2% for the human Smad3–GGCGC complex, 98.4 and 1.6% for the Smad3–GGCT complex and 96.7 and 3.3% for Smad4–GGCCG complex. No structure displays outlier residues. The data were validated using MolProbity^56^.

Figures describing the structures were generated with UCSF Chimera^57^. Structures have been deposited at the PDB: 5MEY, 5MEZ, 5MF0, 5NM9, 5OD6, and 5ODG.

### Isothermal titration calorimetry

ITC measurements were performed using a nano ITC calorimeter (TA Instruments) at 20 °C. DNA and protein samples were dissolved in the same buffer and degassed before the experiments. Concentrations were determined using a NanoDrop system and their predicted extinction coefficients. The NanoAnalyze software (TA Instruments) was used to analyze the binding isotherms. Baseline controls were acquired with buffer and pure DNA solutions. Regarding the stoichiometry of the interactions, palindromic DNAs contain two theoretically equivalent sites (detected in the EMSA assays). However, if the stoichiometry is considered as one of the parameters that can be adjusted together with the *K*_D_, the stoichiometry values obtained were slightly below the expected 0.5, even for the SBE used as control. We assume that these discrepancies are due to unavoidable errors in the determination of the active concentration of duplex DNA present during the titration, and also due to the equilibrium between duplex and single-stranded DNA (some duplexes have melting points close to 37 °C). Fittings were performed using the independent binding sites model. For the GTCT, we obtained Δ*H* of −60 KJ mol^−1^, Δ*S* of −60, and Δ*G* of −42. Similar values were obtained for the GGCT, with −62, −74, and −40, respectively. We obtained Δ*H* of −23, Δ*S* +70, and Δ*G* of −44 for the GGCG.

### Computational methods

We downloaded the mm9 and hg19 human reference genomes using the UCSC^58^ genome Distributed Annotation System server (DAS). From this, we used the EMBOSS^59^ fuzznuc tool to scan for the following DNA patterns: CAGAC, AGAC, GGCCG, GGCTG, GGCGG, and GGCGC. For each of the patterns and from the output of the search, we generated a bed track that were displayed with the IGV 2.3.66 tool^60^, for visual inspection of the results.

The following Gene Expression Ominbus (GEO)^61^ data sets were downloaded for the analysis: (GEO accession numbers ordered as shown in the figures: GSM727561, GSM727586, GSM727564, GSM727589^21^, GSM1266817^23^, GSM727557, GSM727585^21^, GSM539548^9^, GSM761757^22^. With the exception of GSM1266817, all remaining data sets were originally aligned to reference genome hg18. We have referenced them to hg19 using the UCSC liftOver tool^58^.

Additional controls were selected to determine whether other TFs that bind GC-rich sites affect the interaction of Smad proteins with 5GC SBEs (Supplementary Fig. 8). Encyclopedia of DNA Elements (ENCODE) ENCFF484WVM and ENCFF175VXL HepG2 cell data sets (generated by Dr. R. Meyers lab U54HG004576) were downloaded for comparison of Smad4 binding regions in cell lines and for control experiments using the SP1and CTFT transcription factors data. For these controls, we used Smad4 ChIP-Seq peaks that were not overlapped by SP1 ChIP-Seq peaks or by CTCF ChIP-Seq peaks (two different experiments) in human HepG2 cells. Each of the filtered data sets showed similar clustering values than the unfiltered Smad4 HepG2 data set. In addition, to explore the 5GC cluster enrichment outside of promoter regions, we also performed these analyses using all Smad4 ChIP-Seq peaks genome wide. The results still show a notable enrichment of 5GC SBE clusters (18 and 40% of the Smad binding regions have three or more 5GC motifs vs. 6% in the baseline) in hESC and in HepG2 cells, respectively.

We used the GEO data sets GSM1782914^62^ and GSM2746361 for mouse data analysis. The Integrative Genomics Viewer (IGV) was used for the visualization of the data and for generating the maps displayed in several figures^60^.

### Motif analysis

To determine each motif frequency and the corresponding average frequencies and distributions, we created 200 bp DNA regions centered in ChIP-Seq regions that were up to 1000 bp from the transcription starting sites obtaining 1755 distinct regions from Smad1 (GSM1505745), 49 from Smad3 (GSM727585), and 155 from Smad4 (GSM727586). Similar analyses were performed with the Smad4 HepG2 (ENCFF484WVM) cells data set, obtaining 16,463 peaks. We also generated a baseline data set with 500 regions of 200 bp length generated from random human genomic coordinates. We determined the number of CAGAC, AGAC, and GGCGC, GGCTG, GGCCG or GGCGG sites in both ChIP-Seq data and baseline regions.

As additional data sets, we also analyzed 200 bp DNA regions centered in ChIP-Seq peaks genome wide (not restricted to promoters) for SMAD4 data sets GSM727586 (4531 regions) and ENCFF484WVM (45,887 regions). As additional controls, we analyzed peaks in both promoters and genome wide for the SMAD4 data set ENCFF484WVM in which we discarded all peaks that had any overlap with the SP1 data set ENCFF175VXL, obtaining 6393 and 23,084 distinct regions, respectively. The same control was performed discarding overlapped peaks with the CTCF data set ENCFF237OKO, obtaining 9035 and 36,587 distinct regions, respectively.

### Data availability

Smad4 ChIP-Seq data were deposited in the Gene Expression Omnibus database under accession number GSM2746361. NMR assignments and chemical shifts have been deposited in the Biological Magnetic Resonance Data Bank, BMRB entry 26945. Densities and coordinates have been deposited in the Protein Data Bank PDB entries are 5MEY, 5MEZ, 5MF0, 5NM9, 5OD6, and 5ODG^63^.

## Electronic supplementary material

Supplementary Information
