# Supplementary material for: Structural basis for genome wide recognition of 5-bp GC motifs by SMAD transcription factors
Source: Nat Commun. 2017 Dec 12;8:2070. doi: 10.1038/s41467-017-02054-6 (PMC5727232; doi:10.1038/s41467-017-02054-6)
Supplement: Supplementary file 1 — Supplementary Information [file 41467_2017_2054_MOESM1_ESM.pdf]

SUPPLEMENTARY FIGURES

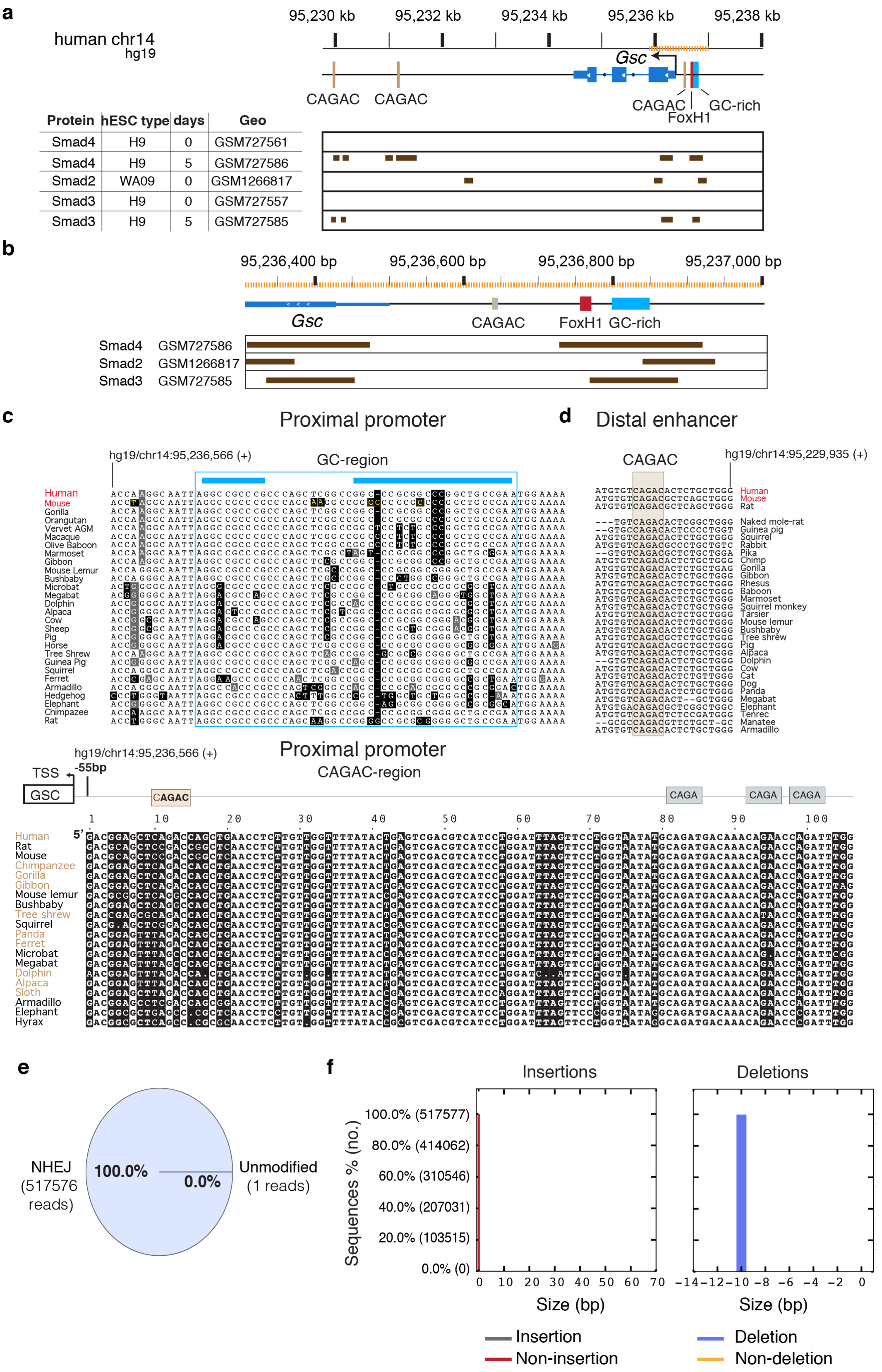

Supplementary Figure 1. Conservation of regulatory regions of *Gsc* in mammals

a. Human ChIP-Seq data analysis of *Gsc* proximal promoter and distal enhancer sites, displaying the GC-rich site, CAGAC and the FoxH1 binding sites boxed**.** Data sets are indicated with the corresponding entries. Coordinates correspond to the human GRCh37/hg19 assembly. The data indicate that Smad2, Smad3 and Smad4 proteins are bound at the GC regions of the *Gsc* promoter.

b. Expanded region of the ChIP-Seq data shown above. Coordinates correspond to the region marked with an orange dashed line in panel **a.**

c. Alignments of the proximal promoter regions of *Gsc* in mammals. Sequence differences in mammals are highlighted with black boxes. The horizontal bars over the GC sequence delimits the original regions described DNaseI footprinting assays *in vitro*, using Smad4 [20]. The CAGAC motif is shown in beige and CAGA sites in grey. The CAGAC site is partially conserved and absent in mouse and rat sequences.

d. Alignment of the conservation of the CAGAC site in the distal enhancer. This CAGAC site is conserved in mammals. Human and mouse are labeled in red.

e. CRISPResso unmodified NHEJ pie chart analysis of clone C1. Quantification of editing frequency as determined by the percentage and number of sequence reads showing unmodified and modified alleles.

f. CRISPResso histogram displaying the size of Clone C1 insertions (left) and deletions (right) [62].

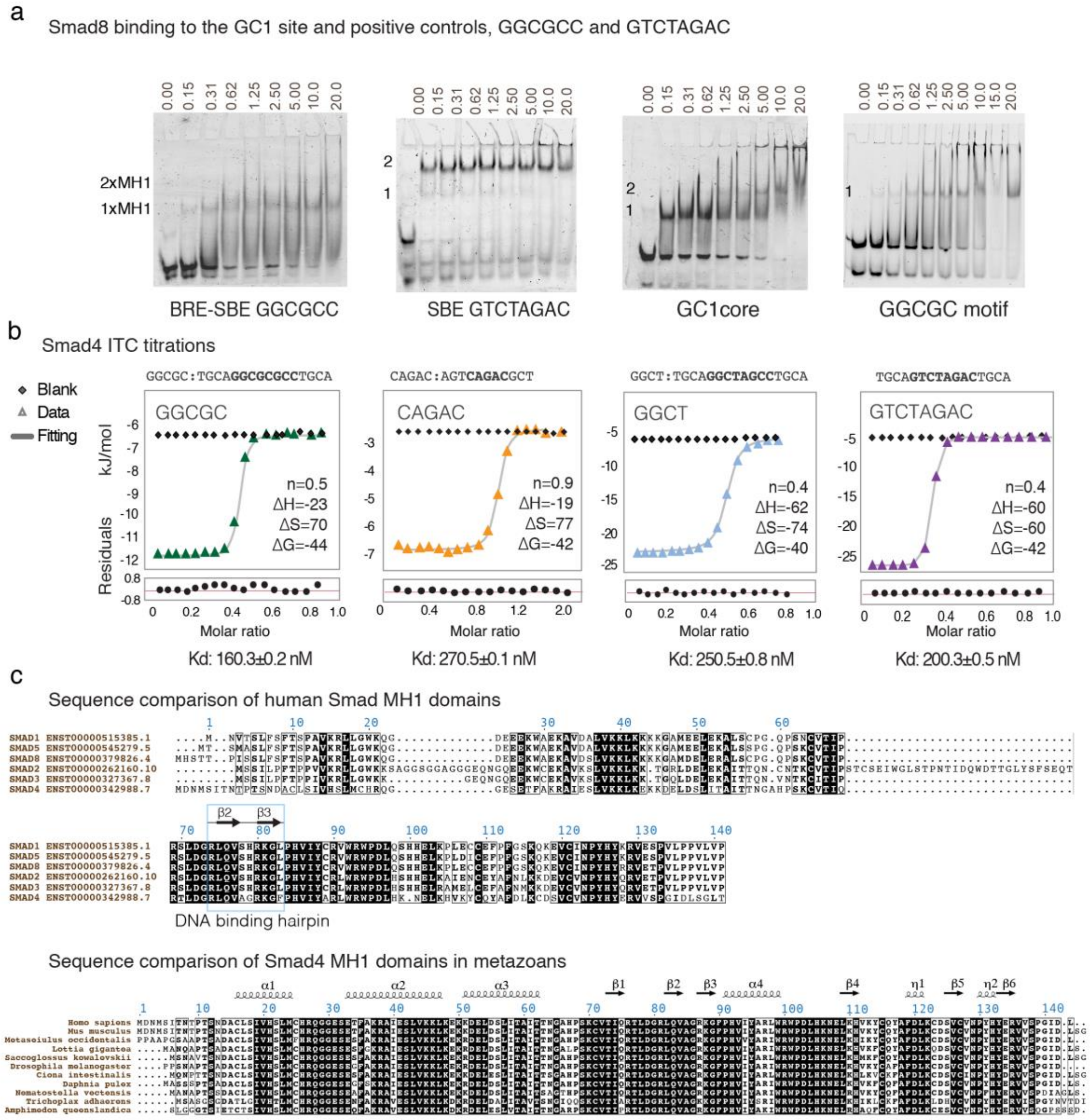

Supplementary Figure 2. Comparison of Smad4 and different R Smad MH1 domains

a. Binding of human Smad8 to GC1-core, the GGCGC motif and to the GTCT and BRE-SBE were analyzed using EMSA assays. Protein concentrations are shown on top of the EMSA (micromolar concentrations).

b. ITC curves and affinity values obtained for the titration of Smad4 MH1 domain with the palindromic GGCGC (green), GTCT (purple) and GGCT sites (blue), and with the native non-palindromic distal enhancer CAGAC site (orange). Data were acquired at 20 °C in Tris buffer, pH 7.0. Sequences are shown on top of the titrations. Data were fitted using the independent model assuming a single binding site.

c. Sequence alignment of the MH1 domains of human Smad1, Smad2 (isoform2), Smad3, Smad4, Smad5 and Smad8 proteins. The DNA binding hairpin observed in the three new GC-complexes of Smad4 is boxed in blue and labeled. Below it, sequence

alignment of the MH1 domain of Smad4 proteins from different organisms. The elements of secondary structure shown on the top of the alignment correspond to the structures of the human sequence.

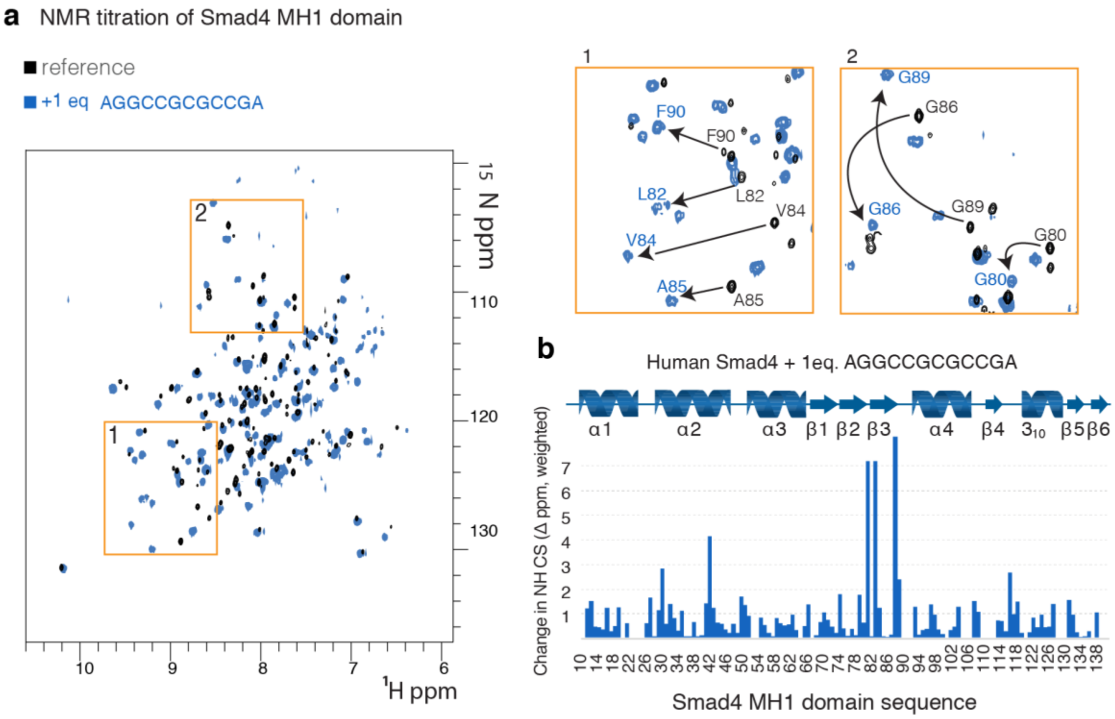

Supplementary Figure 3. NMR titration of Smad4 MH1 domain using HSQC experiments

a. HSQC based titration experiment of Smad4 MH1 (^{15}N-^{2}H labelled, black) with the presence of 1 equivalent of the AGGCCGCGCCGA dsDNA (blue). Several residues are affected upon DNA binding. Two regions of the spectrum are expanded, and the affected residues are labelled.

b. Representation of the chemical shift changes measured for all residues, as in Figure 3c. Represented values have been calculated as described in the methods section.

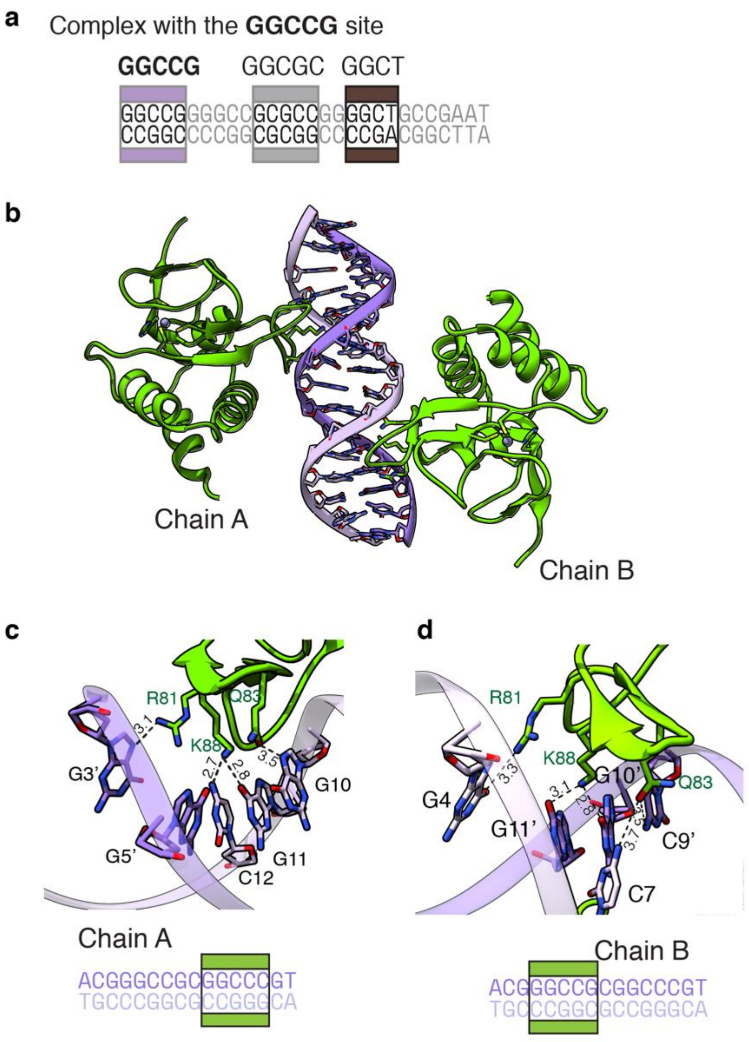

Supplementary Figure 4. Complex of Smad4 MH1 domain with the GGCCG site

a. Schematic representation of the GC1 site with the GGCCG highlighted in violet. The GGCCG DNA used for crystallization was ACGGGCCGCGGCCCGT.

b. Biological assembly of Smad4 MH1 domain, residues 15-138 in complex with the GGCCG site showing one MH1 molecule and the DNA of the Asymmetric Unit, and another MH1 molecule of the neighboring ASU. The binding sites are highlighted with salmon circles.

c. A close view of the chain A binding site is shown. Residues and nucleotides participating in the hydrogen bonds and contacts are displayed and labeled. Distances are shown in Å.

d. A close view of the chain B binding site, as in **c.**

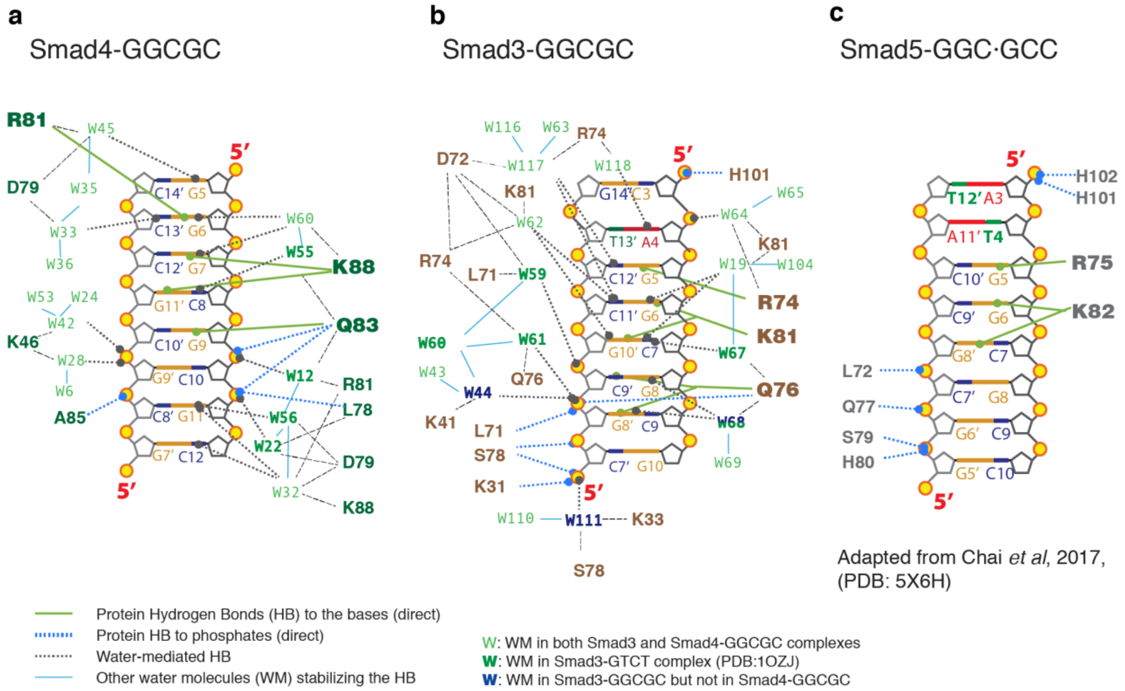

Supplementary Figure 5. Comparison of the contacts present in the GGCGC-Smad3 and Smad4 complexes to that of Smad5 and the GGCGCC site.

a. Summary of specific DNA-protein hydrogen bonds including those mediated by water molecules for the Smad4 GGCGC complex. Water molecules are colored as in figure 4k

b. Summary of specific DNA-protein hydrogen bonds including those mediated by water molecules for the Smad3 GGCGC complex. Water molecules are colored as in figure 4l

c. Representation of the hydrogen bonds as described for Smad5 (PDB: 5X6H), adapted from Chai et al, 2017.

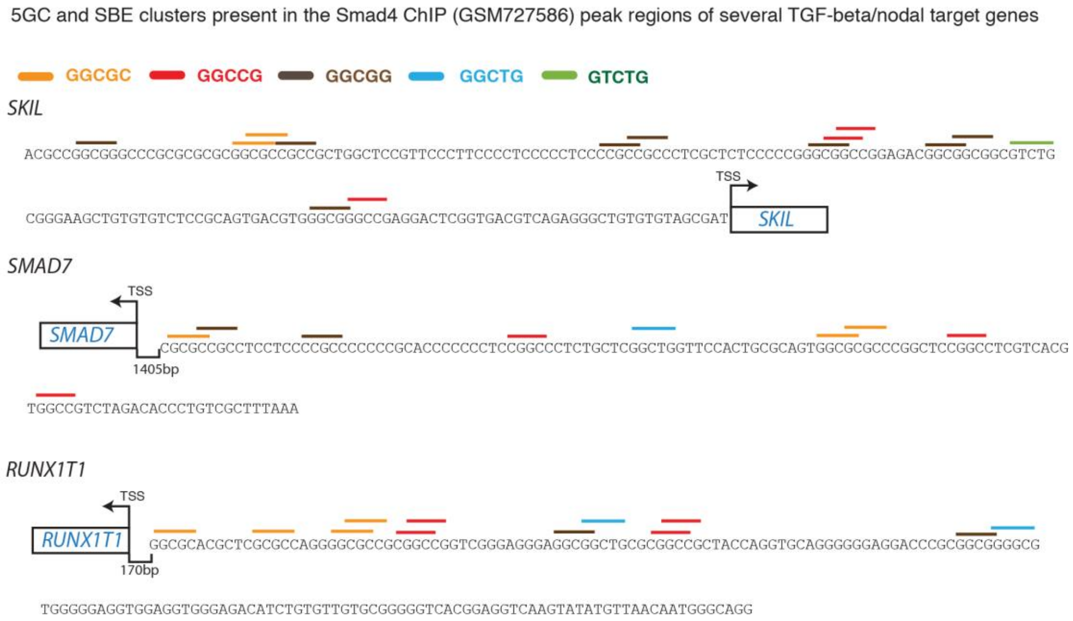

Supplementary Figure 6. New 5GC and CAGAC sites present in nodal/TGF-β stimulated cells in Smad4 ChIP-Seq peaks.

The motifs are indicated as bars, colored in orange, blue, red, brown and green depending on the motif. The three selected examples correspond to promoter regions of the *Skil*, *Smad7* and *RunX1T1* genes.

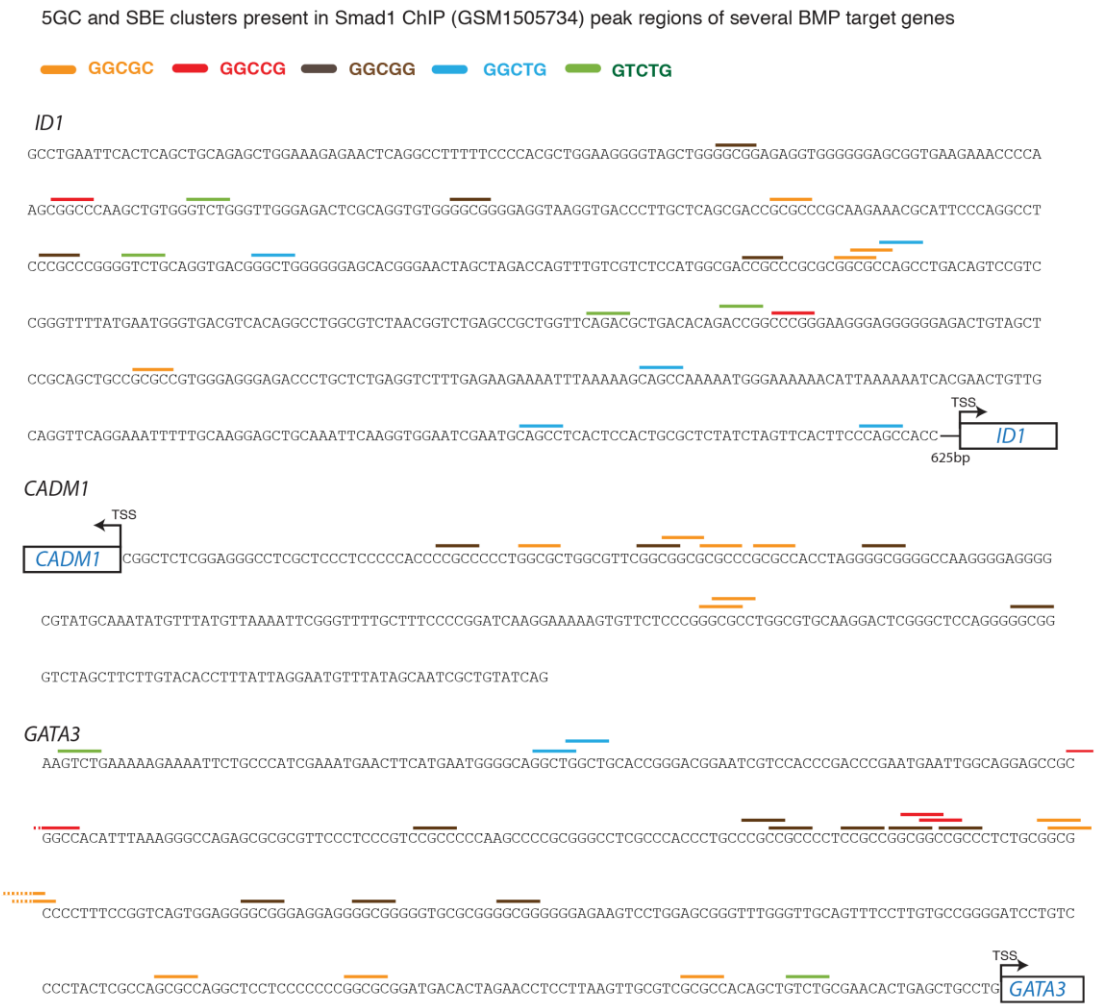

Supplementary Figure 7. New 5GC and CAGAC sites present in BMP-stimulated cells in Smad1 Chip-Seq peaks

The motifs are indicated as bars, colored in orange, blue, red, brown and green depending on the motif. The three selected examples correspond to promoter regions of the *ID1*, *CADM1* and *GATA3* genes suggesting that the 5CG binding motifs are also targets of BMP activated R-Smads.

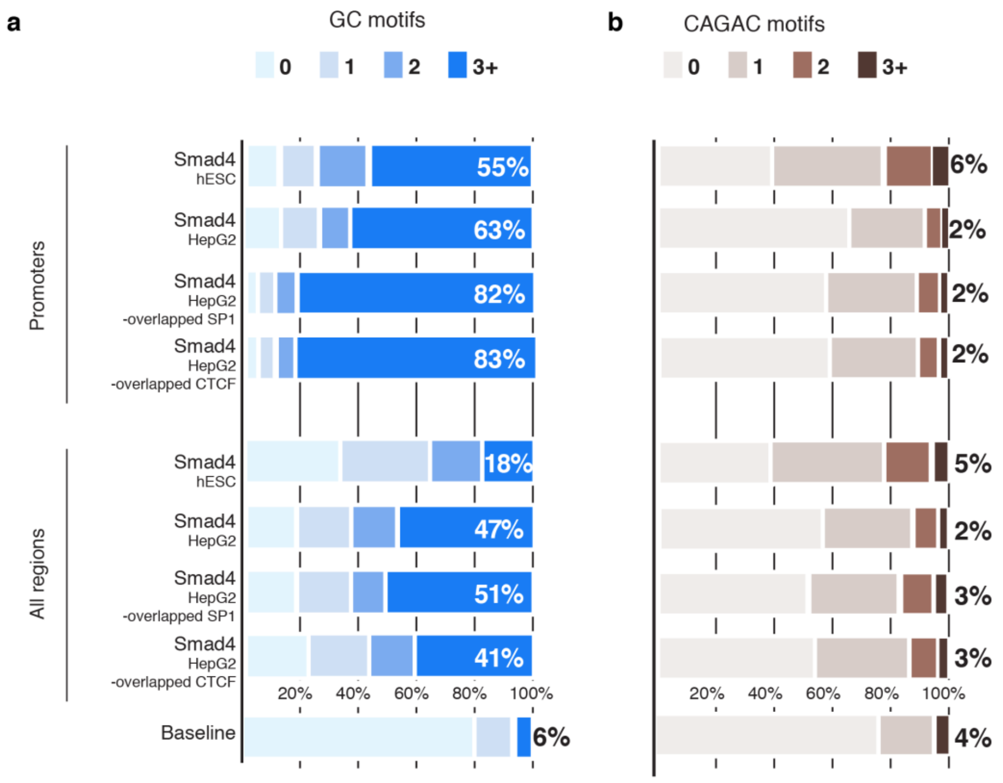

Supplementary Figure 8. The new binding motifs are enriched in nodal/TGF- β and in BMP-stimulated cells in all ChIP-seq regions genome wide

a. Stacked bar-plots showing the number of 5GC motifs per 200bp-region (as %) found in Smad4 bound ChIP-Seq data in ES and in HepG2 cells genome wide expanding the analysis to include all ChIP-seq regions. The result obtained for the promoter site (1000 bp from the TSS) is included for comparison. The overall number of 5GC bound sites is slightly smaller than in the promoter selected data set. Remarkably, clusters of 3+ sites are found more often in promoters than when the analysis includes all ChIP-Seq peaks (coding regions, introns, possible aberrant signals).

We have also analyzed Smad4 promoter bound regions excluding those overlapping with SP1 ChIP-Seq peaks to detect the potential effect Smad4 interaction with a GC-rich TF binder in the selection of Smad4 binding sites. When the ChIP-Seq data of SP1 overlapped with Smad4 peaks is removed, clusters of Smad4 3+ sites are more enriched in 5GC motifs with respect to the total Smad4 bound peaks.

b. The same analysis but focused on CAGAC motifs.

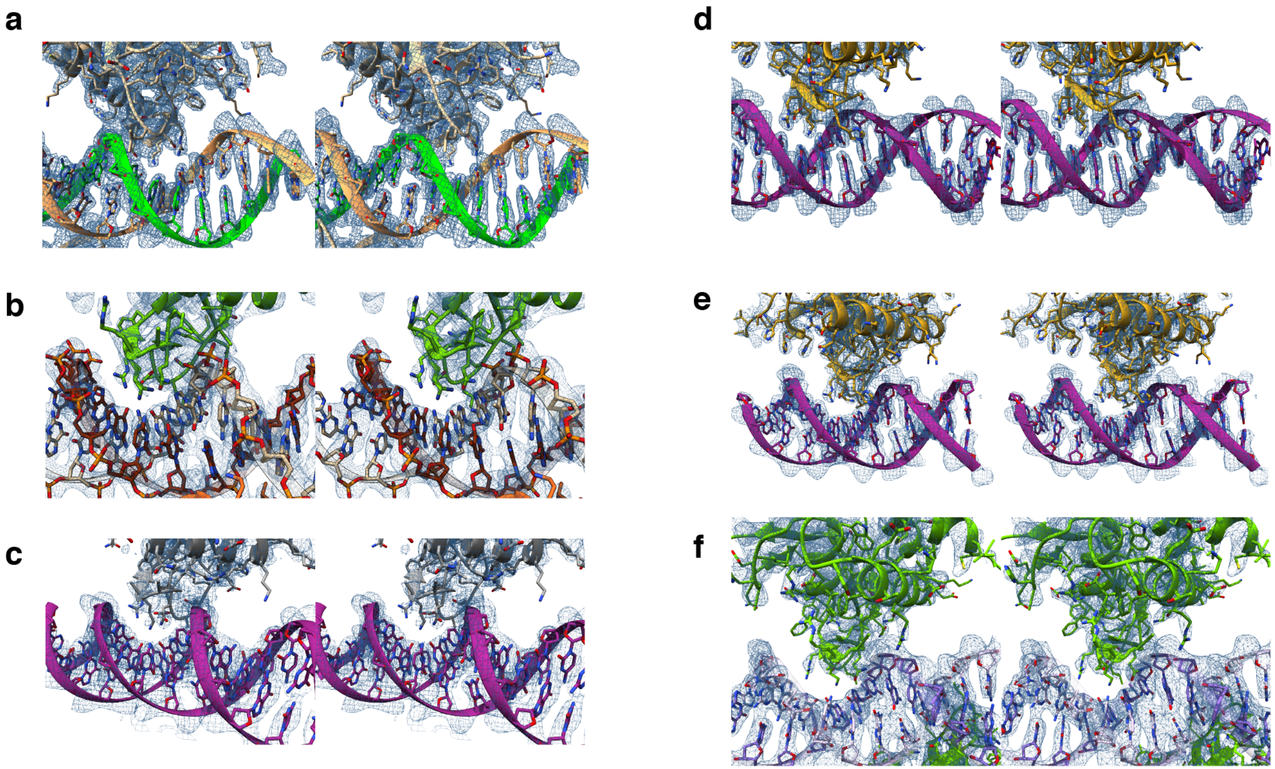

Supplementary Figure 9. Stereo view showing the electron density for the X-ray structures.

All maps shown are contoured at 1σ level (2Fo-Fc)

a. SMAD4-GGCGC (5MEY)

b. SMAD4-GGCT (5MEZ)

c. T_SMAD4-GGCGC (5NM9)

d. SMAD3-GGCGC (5OD6)

e. SMAD3-GGCT (5ODG)

f. SMAD4-GGCCG (5MF0)

SUPPLEMENTARY TABLE 1

Oligonucleotides used in EMSA, in the structural work and in ITC experiments.

EXPERIMENT	NAME	SEQUENCE 5'→3
EMSA	GC1 core	TTAAGGCCGGGGCCGCGCCGGGAATT
	-GGCCGG-	ATTATT-GGCCGG-AAAGCA
	-CCGGGG-	ATTATT-CCGGGG-AAAGCA
	-GGGGCC-	ATTATT-GGGGCC-AAAGCA
	-CCGCGC-	ATTATT-CCGCGC-AAAGCA
	-GCGCCG-	ATTATT-GCGCCG-AAAGCA
	-GCCGGG-	ATTATT-GCCGGG-AAAGCA
	-GGCTGCC-	GTAACCGGCTGCCGAATG
	GC1-Mouse 1	ATTA-CCGCGCCGGGGCTGC-AATC
	GC1-Mouse 2	ATTA-CCGCGCCGGGGC-AATC
	GC2 core	ATTA-GGCCGCCCGCCC-AGCA
	-GGCCGC-	ATTA-GGCCGC-AAAAAAAGCA
	-CCGCCC-	ATTATT-CCGCCC-AAAAAGCA
	-GCCCGC-	ATTATTTT-GCCCGC-AAAGCA
	-CCGCCC-2	ATTATTTTTT-CCGCCC-AGCA
	GC2_30	AACCTGGGCAATTAGGCCGCCCGCCCAGCA
	GC2_14	AACCTGGGCAATTA
	FoxH1	TCTCAATCCACAATC
	SBE	TGCAGTCTAGACTGCA
	BRE-GGCGCC	TGCAGGCGCCTGCA
	CAGAC-DE	TGTGTCAGACGCTCAGCT
**EXPERIMENT**	**NAME**	**SEQUENCE 5'→3**
NMR	NMR-1	GGGGCTGCCGAA
	NMR-2	GGGGCTGCCGAA
	NMR-3	TGCAGTCTAGACTGCA
**EXPERIMENT**	**NAME**	**SEQUENCE 5'→3**
X-RAY	GGCGC-XRAY-Smad4	ATGCGGGCGCGCCCGCAT
	GGCGC-XRAY-Smad3	TGCAGGCGCGCCTGCA
	GGCCG-XRAY	ACGGGCCGCGGCCCGT
	GGCT-XRAY	TGCAGGCTAGCCTGCA

EXPERIMENT	NAME	SEQUENCE 5'→3	Kd (huSmad4) nM
ITC	GGCG	TGCAGGCGCGCCTGCA	160.3±0.2
	GGCT	TGCAGGCTAGCCTGCA	250.5±0.8
	CAGAC	AGTCAGACGCT	270.5±0.1
	GTCT	TGCAGTCTAGACTGCA	200.3±0.5
